# Supplementary material for: A comprehensive review and call for studies on firefly larvae
Source: PeerJ. 2021 Sep 20;9:e12121. doi: 10.7717/peerj.12121 (PMC8459732; doi:10.7717/peerj.12121)
Supplement: Supplemental Information 1 — Abbreviations indicating the main topic of the works are as follows: B, behavior; H, habitat; I, interaction; L, life cycle; M, morphology; P, physiology. [file peerj-09-12121-s001.pdf]

Supplemental Information 1 - Table S1 and references

article: A comprehensive review and call for studies on firefly larvae

authors: William Brent Riley, Simone Policena Rosa and Luiz Felipe Lima da Silveira

Table S1. Synoptic list of references on Lampyridae larva, arranged by subfamily and zoogeographic region, and scored by subject. Abbreviations indicating the main topic of the works: B, behavior; H, habitat; I, interaction; L, life cycle; M, morphology; P, physiology.

Subfamily	Zoogeographic region	Reference	Subject
Cyphonocerinae	Sino-Japanese	Kawashima 2017b	HM
Lamprohizinae	Nearctic	De Cock, Faust & Lewis 2014	B
		Faust & Forrest 2017	BH
		Faust 2017	BM
		Lewis et al. 2020	H
		Schwalb 1961	BL
	Palearctic	Bugnion 1919	M
		Bugnion 1922b	LM
		Bugnion 1929	M
		Korschefsky 1951	M
		Novák 2018a	LM
		Verhoeff 1925	HL
		Vogel 1922	M
Lampyrinae	Nearctic	Buck 1948	MP
		Archangelsky & Branham 1998	M
		Archangelsky & Branham 2001	M
		Boving & Craighead 1931	M
		Branham & Archangelsky 2000	M
		Buschman & Faust 2014	H
		Buschman 1984a	BHL
		Buschman 1988a	BL
		Buschman 1988b	B
		Buschman 2019	B
		Cicero 1994	M
		Day 2011	BIM
		De Cock 2000	BH
		Fabre 1913	B
		Faust 2012	HL
		Faust & Faust 2014	B
		Faust 2010	HL
		Faust 2017	BM
		Gentry 2003	L
		Hess 1920	BHM
		King 1880	L
		LaBella & Lloyd 1991	M
		Lewis et al. 2020	H
		Lloyd 1973a	BI
		Lloyd 1973c	B
		Lloyd 2006	BL
		Lloyd 2018	HM
		Majka & MacIvor 2009	BH
		McDermott 1954	LM
		Murphy & Moiseff 2020	HP
		Newport 1857	L
		Schwalb 1961	BL
		Sivinski 1981	B

Lampyrinae	Nearctic	Sivinski et al. 1998	BHL
		Smedley et al. 2018	P
		Vaz et al. 2021	BHLM
		Wenzel 1896	BM
		Wickham 1895	M
		Wilcox & Lewis 2019	P
		Williams 1917a	BLM
		Williams 1917b	LM
		Wooton 1976	L
		Zaragoza-Caballero et al. 2020	BM
	Neotropical	Barber 1923	M
		Candèze 1861	L
		Costa, Vanin & Casari-Chen 1988	BM
		Nunes et al. 2021	HM
		Schaller 2001	B
		Schaller 2001	B
		Tonolli et al. 2011	M
		Tonolli et al. 2011	M
		Vaz et al. 2021	HM
		Viviani & Bechara 1995	P
		Viviani & Bechara 1995	P
		Viviani 2001	BH
		Viviani 2001	BH
		Viviani et al. 2004	P
		Viviani et al. 2004	P
		Viviani et al. 2008	P
		Viviani et al. 2008	P
		Viviani, Rosa & Martins 2012	LM
		Viviani, Rosa & Martins 2012	LM
	Oriental	Hashmi, Asghar & Hamed 1986	M
		Kusui 1979	HI
		Li et al. 2008	BM
		Lloyd 2018	HM
		Majka & MacIvor 2009	BH
		McDermott 1960	M
		Mobilim & Dawood 2020	H
		Nunes et al. 2021	HM
		Panigrahi 2000	HI
		Qin & Fu 2009	HM
		Raj 1943a	M
		Wu & Perng 2007	H
		Wu & Yang 2008	HI
		Wu et al. 2012	H
		Yoshida et al. 2020	I
	Palearctic	Alvarez & De Cock 1900	HL
		Arvy & Gabe 1949	M
		Beutel 1995	M
		Bongardt 1903	M
		Bongardt 1904	HM
		Boving & Craighead 1931	M
		Bugnion 1922b	LM
		Buschman 1988b	B
		Davydova 1968	M
		Day 2011	BIM
		De Cock & Geisthardt 2007	LM
		De Cock & Matthysen 1999	B
		De Cock & Matthysen 2001	B
		De Cock & Matthysen 2003	B
		De Cock 2004	P
		Dreisig 1974	M
		Fabre 1924	BM
		Fu et al. 2009	BLM

Lampyrinae	Palearctic	Geisthardt 2007	M
		Geisthardt 1979	M
		Gorgadze & Tskhadaia 1995	L
		Gorgadze 1998	L
		Gunn & Gunn 2013	H
		Haddon 1915	HM
		Hadj-Mohammadi & Chaichi 1996	P
		Horne & Horne 2017	L
		Ineichen 2004	BH
		Korschefsky 1951	L
		Lehtonen et al. 2021	B
		Lequet & Faucheux 2015	L
		Lequet & Faucheux 2016	L
		Lewis et al. 2020	H
		Maas et al 2001	MP
		Margry 2013	I
		Meinert 1886	M
		Naisse 1969	P
		Novák 2017	BHL
		Novák 2018b	MP
		O'Donald 1968	I
		Owsjannikow 1864	BP
		Planet 1908	M
		Pototskaja 1983	M
		Rey 1882	M
		Tisi et al. 2014	BMP
		Trice & Tyler 2007	M
		Trice, Tyler & Day 2004	M
		Tyler & Trice 2001	M
		Tyler 1997a	L
		Tyler 1997b	M
		Tyler 2001a	BI
		Tyler 2001b	BIM
		Tyler 2001b	BHILM
		Tyler et al. 2008	P
		Vogel 1912	BM
		Vogel 1915	M
		Wielowiejski 1882	MP
		Wunsch 1995	L
	Panamenian	Lanuza-Garay et al. 2020	BI
		Madruga & Branham 2020	HLM
		Madruga 2018	BI
		Madruga-R. & Hernández-Q. 2010	BI
		Vaz et al. 2021	BHLM
		Zaragoza-Caballero et al. 2020	BM
	Saharo-Arabian	Bugnion 1933	M
		Bugnion 1934	M
		Cros 1924	B
		Lheritier 1955	BI
	Sino-Japanese	Bessho-Uehara, Konishi & Oba 2017	P
		Fang et al. 2013	IP
		Fu & Meyer-Rochow 2013	BLM
		Hayashi 1991	M
		Kawashima & Takai 2004	M
		Kawashima 2017a	M
		Kawashima 2019a	M
		Ohba 2005	BI
		Ohba 2007	B
		Ohba, Goto & Kawashima 1995	M
		Sato 2019	B
		Wang et al 2007	BL
		Yiu 2011	M

Luciolinae	Afrotropical	Imms 1933	M
		Kaufmann 1965	HM
	Australian	Armitage 1908	BM
		Ballantyne 2001	M
		Ballantyne & Lambkin 2000	M
		Ballantyne & Lambkin 2009	M
		Ballantyne & Lambkin 2013	M
		Ballantyne & McLean 1970	M
		Ballantyne 1968	M
		Ballantyne 1987	HM
		Ballantyne 1988	HM
		Ballantyne et al. 2019	M
	Madagascan	Fairmaire 1900	M
	Oceanian	Ballantyne & Buck 1979	BHM
		Ballantyne & Lambkin 2009	M
		Ballantyne & Lambkin 2013	M
		Ballantyne & McLean 1970	M
		Ballantyne 1968	M
		Ballantyne et al. 2019	M
		Buck & Buck 1970	BH
		Deheyn & Ballantyne 2009	M
		Fu, Ballantyne & Lambkin 2012a	M
		Lloyd 1973b	H
		Saxton et al. 2019	H
	Oriental	Annadale 1900	BH
		Annandale 1904	BH
		Annandale 1906	BH
		Ballantyne & Lambkin 2006	M
		Ballantyne & Lambkin 2009	M
		Ballantyne & Lambkin 2013	M
		Ballantyne & Menayah 2002	M
		Ballantyne et al. 2013	M
		Ballantyne et al. 2019	M
		Bertrand 1973	M
		Blair 1927	M
		Chanchay et al. 2019	P
		Chen & Chen 1997	L
		Chen et al. 2012	BIM
		Chen et al. 2018	P
		Chen et al. 2021	P
		De Maria, Pellegrino & Okabe 1967	BI
		Fletcher 1919	HM
		Fu & Ballantyne 2009	M
		Fu et al. 2009	BLM
		Fu, Seelan & Dawood 2017	I
		Ganguly & Gosh 1982	BM
		Gardner 1946	M
		Hasama 1942a	MP
		Hasama 1942b	MP
		Ho & Chiang 1997	H
		Ho & Chiang 2002	H
		Ho & Huang 2003	P
		Ho & Jong 1997	HM
		Ho & Su 2000	M
		Ho 2002	BHM
		Ho 2004	HL
		Ho et al. 2010	BM
		Ho et al. 2014	BI
		Ho, Chiang & Yang 2006	L
		Ho, Chu & Chu 1998	H
		Ho, Fang & Yang 2014	L
		Ho, Su & Huang 2003	L

Luciolinae	Oriental	Jakla, Thancharoen & Pinkaew 2020	L
		Jeng et al. 1998	M
		Jeng, Lai & Yang 2003	HM
		Kanjana 2017	M
		Lewis et al. 2020	H
		Liew & Schilthuizen 2014	I
		Lloyd, Wing & Hongtrakul 1989	H
		Loomboot et al. 2007	BL
		Mehta 1932	LM
		Nada, Ballantyne & Jusoh 2021	BHM
		Oba, Oba & Konishi 2012	H
		Ohba & Sim 1994	BLM
		Raj 1947	M
		Raj 1952	BHIM
		Thancharoen et al. 2007	HLM
		Vongsangnak, Chumnanpuen & Sriboonlert 2016	P
		Wu & Yang 2008	HI
		Wu et al. 2012	H
		Yeh 1999	BHI
	Palearctic	Bugnion 1922a	M
		Ballantyne & Lambkin 2009	M
		Bourgeois 1884	M
		Bugnion 1921	LM
		Fu & Ballantyne 2009	M
		Fu, Ballantyne & Lambkin 2012b	M
		Fu, Ballantyne & Lambkin 2012b	M
		Ghigi 1901	M
		Jeng, Lai & Yang 2003	HM
		Lee & Boo 1991	M
		Schaller & Schwalb 1961	B
		Tozzetti 1866	MP
		Tozzetti 1870	MP
		Tyler & Trice 2001	M
	Sino-Japanese	Ballantyne & Lambkin 2009	M
		Ballantyne & Lambkin 2013	M
		Ballantyne et al. 2011	HM
		Ballantyne et al. 2013	M
		Ballantyne et al. 2016	BM
		Ballantyne et al. 2019	M
		Bessho-Uehara & Oba 2017	P
		Fallon et al 2018	P
		Fu & Ballantyne 2006	M
		Fu & Ballantyne 2008	HM
		Fu & Ballantyne 2009	M
		Fu & Meyer-Rochow 2012	BM
		Fu & Meyer-Rochow 2021	P
		Fu 2009	B
		Fu et al. 2005a	BLM
		Fu et al. 2005b	B
		Fu et al. 2006a	B
		Fu et al. 2006b	BLM
		Fu et al. 2007	M
		Fu et al. 2009	BLM
		Fu, Ballantyne & Lambkin 2010	M
		Fu, Ballantyne & Lambkin 2012a	M
		Fu, Ballantyne & Lambkin 2012b	M
		Fu, Ohba & Lei 2004	ILM
		Fu, Wang & Lei 2005	BM
		Gotou et al. 2005	H
		Hanneda 1977	L
		Hara 1962	M
		Hasama 1942b	MP

Luciolinae	Sino-Japanese	Hatano & Kato 1963	P
		Hayashi 1991	M
		Imuta, Nakamura & Hirata 1994	B
		Jeng, Lai & Yang 2003	HM
		Kakehashi, Kuranishi & Kamata 2013	H
		Kakehashi, Kuranishi & Kamata 2014	B
		Kanda 1934	L
		Katsuno 1963	L
		Katsuno 1968	L
		Kawashima 2018	M
		Kawashima 2019b	M
		Kawashima 2020a	M
		Kawashima 2020b	M
		Kawashima et al. 2016	M
		Kiichiro 1961	BH
		Kim et al. 2003	L
		Kim, Kwon & Suh 2008	L
		Kondo & Tanaka 1989	I
		Kondo & Yagi 2007	HI
		Kumode, Tanaka & Yuma 1999	L
		Kyuka et al. 2010	H
		Lee et al. 2003	L
		Lee et al. 2008	P
		Lewis et al. 2020	H
		Matsuda et al. 2010	H
		Mei et al. 2020	L
		Minami 1966	L
		Moriya, Yamagauchi & Nakagoshi 2009b	L
		Moriya, Yamauchi & Nakagoshi 2006	B
		Moriya, Yamauchi & Nakagoshi 2007	M
		Moriya, Yamauchi & Nakagoshi 2009a	L
		Nakane & Ohba 1981	L
		Natsumeda, Matsuda & Yuma 2013	H
		Nishijima, Yasuoka & Maeto 2010	I
		Noh et al. 1990	L
		Oba et al. 2006	P
		Oba et al. 2010	P
		Oh et al. 2009a	L
		Oh et al. 2009b	B
		Ohba & Goto 1989	BM
		Ohba & Goto 1991	HLM
		Ohba 1983	HM
		Ohba 1986	L
		Ohba 1988a	L
		Ohba 1988b	BH
		Ohba 1991	L
		Ohba 1997	L
		Ohba et al. 1994	BLM
		Ohtsuki et al 2014	P
		Okada 1928	BILM
		Sekine et al. 2007	H
		Tabaru et al. 1970	IP
		Takeda et al. 2006	H
		Yajima 2007	L
		Yajima 2015	L
		Yang et al. 2020	P
		Yiu 2011	M
		Yuan et al. 2007	M
		Yuma 1981b	BM
		Yuma 1982	B
		Yuma 1984	L
		Yuma 1986	P

Luciolinae	Sino-Japanese	Yuma 2007	H
		Zhang et al. 2019	P
		Zhang et al. 2020	P
		Zhang et al. 2021	P
		Zheng et al. 2008	B
		Zheng et al. 2008a	BMP
Ototretinae	Oriental	Zheng et al. 2008b	B
Ototretinae	Sino-Japanese	Ho 2002	BHL
		Hayashi 1991	L
		Kawashima & Satou 2004	BH
		Ohba 1983	HM
		Yiu 2011	M
	unspecified	Ohba, Goto & Kawashima 1996	M
Photurinae	Nearctic	Janisova & Bocakova 2013	M
		Boving & Craighead 1931	M
		Buck & Case 1961	P
		Buck, Case & Hanson 1963	P
		Buschman 1984b	BHL
		Buschman 2019	B
		Carlson & Evans 1986	P
		Carlson & Jalenak 1986	P
		Carlson 1965	P
		Carlson 1968a,b	P
		Carlson 1972	P
		Christensen & Carlson 1981	P
		Christensen & Carlson 1982	P
		Christensen et al. 1983	P
		Cicero 1982	M
		Cicero 1994	M
		Copeland 1981	P
		Domagala & Ghiradella 1984	M
		Faust & Faust 2014	B
		Faust 2017	BM
		Forsyth, Peterle & Bandy 1983	IP
		Hastings & Buck 1956	P
		Hess 1922	M
		Keiper & Solomon 1972	B
		LaBella & Lloyd 1991	M
		Lewis et al. 2020	H
		Lloyd 1973a	BI
		Lloyd 1973c	B
		Lloyd 2018	HM
		McLean, Buch & Hanson 1972	B
		Murphy & Moiseff 2019	M
		Nathanson & Hunnicutt 1979	P
		Oertel & Case 1976	P
		Oertel, Linberg & Case 1975	M
		Owens & Lewis 2021	BL
		Peterson 1970	M
		Stansbury & Moczek 2014	MP
		Strause & Case 1981	P
		Strause & DeLuca 1981	P
		Strause, DeLuca & Case 1979	P
		Underwood, Tallomy & Pesek 1997	M
		Vencl et al. 2012	HP
		Wienhausen & DeLuca 1985	P
		Williams 1914	LMP
		Williams 1917a	BLM
		Williams 1917b	LM
	Neotropical	Candèze 1861	L
		Costa, Vanin & Casari-Chen 1988	BM
		Rosa 2007	LM

Photurinae	Neotropical	Viviani 2001	BH
		Wynberg et al. 1980	P
Psilocladinae	Neotropical	Kok, Doorn & Dezfoulian 2019	BI
		Vaz, Silveira & Rosa 2020	HM
Pterotinae	Nearctic	Sivinski 1981	B
		Dean 1979	BHM
Incertae sedis	Oriental	Peterson 1957	BI
		Bess 1956	BHILM
		Bibi & Ittichan 2005	I
		Blair 1928	M
		Brues 1941	M
		Ho 2002	BHL
		Hutson & Austin 1924	BL
		Jeng et al. 2021	L
		Kanjana et al. 2017	M
		Lucas 1904	M
		Mbugua, Wong & Ratnayeke 2020	B
		Paiva 1919	BIM
		Raj 1941	M
		Raj 1943b	L
		Raj 1943c	M
	Sino-Japanese	Wijekoon, Wegiriya & Bogahawatta 2016	I
		Yiu 2011	M
unspecified	Afrotropical	Yiu 2011	BL
	Australian	Sodeman, Rodrick & Vincent 1980	BI
	unspecified	Robinson 2009	I
		Harvey & Hall 1929	P

References

Álvarez JG, & De Cock, R. 2011. The biology and distribution of glow-worms (Coleoptera: Lampyridae) in Spain. In Day JC, ed. *Lampyrid: The Journal of Bioluminescent Beetle Research Volume 1.* Oxfordshire: Brazen Head Publishing, 22–31.

Amaral DT, Silva JR, Viviani VR. 2017. Transcriptomes from the photogenic and non-photogenetic tissues and life stages of the *Aspisoma lineatum* firefly (Coleoptera: Lampyridae): Implications for the evolutionary origins of bioluminescence and its associated light organs. *Gene Reports* **8**:150–159 DOI: 10.1016/j.genrep.2017.07.004

Annandale N. 1900. Observations on the habits and natural surroundings of insects made during the 'Skeat expedition' to the Malay peninsula, 1899–1900. VI. Insect Luminosity. An aquatic lampyrid larva. *Proceedings of the Zoological Society of London* **1900**:862–865.

Annandale N. 1904. The occurrence of an aquatic glow-worm in India. *Proceedings of the Asiatic Society of Bengal* **1904**:82–83.

Annandale N. 1906. 16. Notes on the freshwater fauna of India No. III. - An Indian aquatic cockroach and beetle larva. *Journal of the Asiatic Society of Bengal* **II(4)**:105–107.

Archangelsky M & Branham MA. 1998. Description of the preimaginal stages of *Pyractomena borealis* (Randall, 1838) (Coleoptera: Lampyridae), and notes on its biology. *Proceedings of the Entomological Society of Washington* **100**:421–430.

Archangelsky M 2010. Larval and pupal morphology of *Pyractonema nigripennis* Solier (Coleoptera: Lampyridae: Photinini) and comparative notes with other Photinini larvae. *Zootaxa* **2601**: 27–44.

Archangelsky M, Branham M. 2001. Description of last instar and pupa of *Pyropyga nigricans* (Coleoptera: Lampyridae, Photinini) and comparison with larvae of other Photinini genera. *The Canadian Entomologist* **33**(2): 155–164 DOI: 10.4039/Ent133155-2.

Archangelsky M. 2004. Description of the last larval instar and pupa of *Aspisoma fenestrata* Blanchard, 1837 (Coleoptera: Lampyridae) with brief notes on its biology. *Tijdschrift voor Entomologie* **147**:49–55.

Armitage RW. 1908. Notes on the Queensland firefly beetle, *Luciola flavicollis*. *Victorian Naturalist* **XXV**:28–30.

Arvy L, Gabi M. 1949. Données histologiques sur l'organe photogène chez la larve de *Pelania mauritanica* L. *Annales des Sciences Naturelles Zoologie* **11**:263–268.

Ballantyne L, Fu X, Lambkin C, Jeng M-L, Faust L, Wijekoon WMCD, Li D, Zhu T. 2013. Studies on South-east Asian fireflies: *Abscondita*, a new genus with details of life history, flashing patterns and behaviour of *Abs. chinensis* (L.) and *Abs. terminalis* (Olivier) (Coleoptera: Lampyridae: Luciolinae). *Zootaxa* **3721**(1):1–048 DOI: 10.11646/zootaxa.3721.1.1

Ballantyne L.A. 1988. The identities of *Luciola australis* (F.) and *L. guerini* Laporte (Coleoptera: Lampyridae). *Journal of the Australian Entomological Society* **27**:161–165.

Ballantyne L.A. 2001. A redescription and reassignment of *Luciola guerini* Ballantyne (Coleoptera: Lampyridae: Luciolinae). *Australian Entomologist* **27**(4):117–123.

Ballantyne LA, Buck E. 1979. Taxonomy and behavior of *Luciola* (*Luciola*) *aphrogeneia*, a new surf firefly from Papua New Guinea. *Transactions of the American Entomological Society* **105**:117–137.

Ballantyne LA, Fu XH, Shih CH, Cheng CY & Yiu V. 2011. *Pteroptyx maipo* Ballantyne, a new species of bent-winged firefly (Coleoptera: Lampyridae) from Hong Kong, and its relevance to firefly biology and conservation. *Zootaxa* **2931**:8–34 DOI: 10.11646/zootaxa.2931.1.2.

Ballantyne LA, Lambkin C. 2006. A phylogenetic reassessment of the rare S. E. Asian firefly genus *Pygoluciola* Wittmer (Coleoptera: Lampyridae: Luciolinae). *Raffles Bulletin of Zoology* **54(1)**:21–48.

Ballantyne LA, Lambkin C. 2009. Systematics of Indo-Pacific fireflies with a redefinition of Australasian *Atyphella* Olliff, Madagascan *Photuroluciola* Pic, and description of seven new genera from the Luciolinae (Coleoptera: Lampyridae). Zootaxa 1997:1–188 DOI: 10.11646/zootaxa.1997.1.1.

Ballantyne LA, Lambkin CL, Ho J-Z, Jusoh WFA, Nada B, Nak-Eiam S, Thancharoen A, Wattanachaiyingcharoen W, Yiu V. 2019. The Luciolinae of S. E. Asia and the Australopacific region: a revisionary checklist (Coleoptera: Lampyridae) including description of three new genera and 13 new species. *Zootaxa*: **4687(1)**: 1-174. DOI: 10.11646/zootaxa.4687.1.1

Ballantyne LA, Lambkin CL, Luan X, Boontop Y, Nak-Eiam S, Pimpasalee S, Silalom S, Thancharoen A. 2016. Further studies on south eastern Asian Luciolinae: 1. *Sclerotia* Ballantyne, a new of fireflies with back swimming larvae 2. *Triangulara* Pimpasalee, a new genus from Thailand (Coleoptera: Lampyridae). *Zootaxa* **4170**(2): 201–249 DOI: 10.11646/zootaxa.4170.2.1

Ballantyne LA, Lambkin CL. 2013. Systematics and phylogenetics of Indo-Pacific Luciolinae fireflies (Coleoptera: Lampyridae) and the description of new genera. *Zootaxa* **3653**:1–162 DOI: 10.11646/zootaxa.3653.1.1

Ballantyne LA, McLean MR. 1970. Revisional studies on the firefly genus *Pteroptyx* Olivier (Coleoptera: Lampyridae: Luciolinae: Luciolini). *Transactions of the American Entomological Society* **96**:223–305.

Ballantyne LA, Lambkin C. 2000. The Lampyridae of Australia (Coleoptera: Lampyridae: Luciolinae: Luciolini). *Memoirs of the Queensland Museum* **46(1):**15–93.

Ballantyne LA, Menayah R. 2002. A description of larvae and redescription of adults of the firefly *Pteroptyx valida* Olivier in Selangor, Malaysia (Coleoptera: Lampyridae: Luciolinae), with notes on Luciolinae larvae. *Raffles Bulletin of Zoology* **50(1)**:101–109.

Ballantyne LA. 1968. Revisional Studies of Australian and Indomalayan Luciolini (Coleoptera, Lampyridae, Luciolinae). *University of Queensland Papers, Department of Entomology, University of Queensland Press* **II(6)**:103–139.

Ballantyne LA. 1987. Lucioline Morphology, Taxonomy and Behaviour: A Reappraisal. (Coleoptera, Lampyridae). *Transactions of the American Entomological Society* **113**:171–188.

Barber HS. 1923. A remarkable wingless glow-worm from Ecuador (Coleoptera, Lampyridae). *Insecutor Inscitiae Menstruus* **11**(10–12):191–194.

Bertrand HPI. 1973. Part X1. Larvae and Pupae of Water Beetles collected from the Island of Ceylon. *Bulletin of the Fisheries Research Station, Sri Lanka (Ceylon)* **24(1/2):**95–112.

Bess HA. 1956. Ecological notes on *Lamprophorus tenebrosus* (Walker)(Coleoptera: Lampyridae), an enemy of the giant African snail. *Proceedings, Hawaiian Entomological Society* **16(1)**:24–29.

Bessho-Uehara M, Konishi K, Oba Y. 2017. Biochemical characteristics and gene expression profiles of two paralogous luciferases from the Japanese firefly *Pyrocoelia atripennis* (Coleoptera, Lampyridae, Lampyrinae): insight into the evolution of firefly luciferase genes. *Photochemical & Photobiological Sciences* **16**(8):1301–1310. DOI: 10.1039/c7pp00110j

Bessho-Uehara M, Oba Y. 2017. Identification and characterization of the Luc2-type luciferase in the Japanese firefly, *Luciola parvula*, involved in a dim luminescence in immobile stages. *Luminescence* **32**(6):924–931 DOI: 10.1002/bio.3273

Beutel RG. 1995. Phylogenetic analysis of Elateriformia (Coleoptera: Polyphaga) based on larval characters. *Journal of Zoological Systematics and Evolutionary Research* **33**(2):145–171 DOI: 10.1111/j.1439-0469.1995.tb00222.x

Bibi R, Ittichan L. 2005. Feeding behavior and prey size selection of a glow worm (*Lamprigera* sp). In Harrison RD ed. *Proceedings of the CTFS-AA International Field Biology Course 2005.* Khao Chong, Center for Tropical Forest Science, 92–94.

Blair K. 1927. An aquatic lampyrid larva from S. Celebes. *Ecological Entomology* **75(1)**:43–45 DOI: 10.1111/j.1365-2311.1927.tb00056.x

Blair KG. 1928. Results of an expedition to Korinchi Peak, Sumatra. XII: Coleoptera Serricornia (Teredilia, Malacodermata and Buprestidae) collected in Korinchi, West Sumatra, by Messrs. H. C. Robinson and C. Boden Kloss. *Journal of the Federated Malay States Museums* **8**:175–184.

Bongardt J. 1903. Beitrage zur Kenntnis der Leuchtorgane einheimischer Lampyriden. *Zeitschrift für wissenschaftliche Zoologie* **75**:1–45.

Bongardt J. 1904. Zur Biologie unserer Leuchtkäfer. *Naturwissenschaftliche Wochenschrift* **19**:305–310.

Bourgeois J. 1884. Cébrionides, Dascillides, Malacodermes. In: Fauvel A., ed. *Faune gallo-rhénane ou species des insects que habitant la France, la Belgique, la Hollande, le Luxenbourg, la Prusse Rhénane, le Nassau et le Valais. Tome IV [1884–1894], Coléoptères.* Caen: F. Le Blanc-Hardel, 1–208

Boving AG, Craighead FC. 1931. *Synopsis of the principal larval forms of the order Coleoptera.* Brooklyn, New York: Brooklyn Entomological Society.

Branham MA, Archangelsky M. 2000. Description of the last larval instar and pupa of *Lucidota atra* (G. A. Olivier 1790) (Coleoptera: Lampyridae), with a discussion of abdominal segment homology across life stages. *Proceedings of the Entomological Society of Washington* **102:**869–877.

Brues CT. 1941. Characteristics of the larviform female of the lampyrid beetle, *Lamprophorus*. *Psyche* **48:**41–44 DOI: 10.1155/1941/60591.

Buck E, Buck JB. 1970. A firefly of the spray zone. In *Alpha Helix Research Program: 1969-1970.* San Diego: University of California, 16.

Buck J, Case JF, Hanson FE. 1963. Control of flashing in fireflies. III. Peripheral excitation. *Biological Bulletin* **125**(2):251–269. DOI 10.2307/1539401

Buck J, Case JF. 1961. Control of flashing in fireflies. I. The lantern as a neuroeffector organ. *Biological Bulletin* **121**(2):234–256 DOI 10.2307/1539429

Buck JB. 1948. The anatomy and physiology of the light organ in fireflies. *Annals of the New York Academy of Sciences* **49**:397–482 DOI: 10.1111/j.1749-6632.1948.tb30944.x

Bugnion E. 1919. Les insectes phosphorescents. *Bulletin de la Sociétè Murithienne* **39**: 82–124.

Bugnion E. 1921. La biologie delà Luciole (Luciola lusitanica). *Revue d'Histoire Naturelle Appliquée* **1**:1–7.

Bugnion E. 1922a. La larve de la Luciole (Luciola lusitanica Charp.). *Annales des Sciences Naturelles Zoologie* **5:** 29–59.

Bugnion E. 1922b. Etudes relatives à l'anatomie et à l'embryologie des vers luisants ou Lampyrides. *Bulletin Biologique de la France et de la Belgique* **56**:1–5.

Bugnion E. 1929. *Le ver-luisant provençal et la luciole niçoise. Mémoire, supplément au "Riviera Scientifique" (année 1929).* Nice: Association Tipographique.

Bugnion E. 1933. Les papilles caudales du grand lampyre algerien *Pelania mauritanica*. *Bulletin Biologique de la France et de la Belgique* **67**:461–473

Bugnion E. 1934. La larve du grand lampyre Algérien (*Pelania mauritanica* L.) Biologie, Anatomie, Physiologie. *Revue Suisse de Zoologie* **41(40):**699–733.

Buschman LL, Faust LF. 2014. Lampyrids recovered from emergence traps in the Great Smoky Mountains National Park. *Journal of the Kansas Entomological Society* **87**(2):245–248 DOI: 10.2317/JKES130409.1

Buschman LL. 1984a. Biology of the firefly *Pyractomena lucifera* (Coleoptera: Lampyridae). *Florida Entomologist* **67**:529–542.

Buschman LL. 1984b. Larval Biology and Ecology of *Photuris* Fireflies (Lampyridae: Coleoptera) in Northcentral Florida. *Journal of the Kansas Entomological Society* **57(1):** 7–16.

Buschman LL. 1988a. Larval development and its photoperiodic control in the firefly *Pyractomena lucifera* (Coleoptera: Lampyridae). *Annals of the Entomological Society of America* **81**(1) 82–90. DOI: 10.1093/aesa/81.1.82

Buschman LL. 1988b. Light organs of immature fireflies (Coleoptera: Lampyridae) as eye-spot/false-head displays. *The Coleopterists Bulletin* **42(1)**:94–97.

Buschman LL. 2019. *Insects of Western North America 11. Bioluminescent behavior of North American firefly larvae (Coleoptera: Lampyridae) with a discussion of function and evolution.* C.P. Fort Collins: Gillette Museum of Arthropod Diversity, Colorado State University. Available at https://mountainscholar.org/handle/10217/194307

Campos SVN, Silveira LFL, Mermudes JRM. 2018. Systematic review of the giant firefly *Cratomorphus cossyphinus*: sexual dimorphism, immature stages and geographic range (Coleoptera: Lampyridae). *Annales Zoologici* **68**(1):57–84. DOI: 10.3161/00034541ANZ2018.68.1.003

Carlson AD, Evans PD. 1986. Inactivation of octopamine in larval firefly light organs by a high-affinity uptake mechanism. *Journal of Experimental Biology* **122**: 369–385 DOI: 10.1242/jeb.122.1.369

Carlson AD, Jalenak M. 1986. Release of octopamine from the photomotor neurones of the larval firefly lanterns. *Journal of Experimental Biology* **122**: 453–457 DOI: 10.1242/jeb.122.1.453

Carlson AD. 1965. Factors affecting firefly larval luminescence. *The Biological Bulletin* **129(2):**234–243.

Carlson AD. 1968a. Effect of drugs on luminescence in larval fireflies. *Journal of Experimental Biology* **49:**195–199.

Carlson AD. 1968b. Effect of adrenergic drugs on the lantern of the larval *Photuris* firefly. *Journal of Experimental Biology* **48:**381–387.

Carlson AD. 1972. Comparison of transmitter and synephrine on luminescence induction in the firefly larva. *Journal of Experimental Biology* **57**(3):737–743 DOI 10.1242/jeb.57.3.737

Carvalho MC, Tomazini A, Amaral DT, Murakami MT, Viviani VR. 2020. Luciferase isozymes from the Brazilian *Aspisoma lineatum* (Lampyridae) firefly: origin of efficient pH-sensitive lantern luciferases from fat body pH-insensitive ancestors. *Photochemical and Photobiological Sciences* **19**(12):1750–1764 DOI: 10.1039/D0PP00272K

Chanchay P, Vongsangnak W, Thancharoen A, Sriboonlert A. 2019. Reconstruction of insect hormone pathways in an aquatic firefly, *Sclerotia aquatilis* (Coleoptera: Lampyridae), using RNA-seq. *PeerJ* **7**:e7428 DOI: 10.7717/peerj.7428

Chen CT, Wu CH, Jeng ML, Yang PS. 2012. Chemical defense of aquatic larvae of the firefly (*Aquatica ficta*): microanatomy of eversible organs, chemicals of glandular secretion, and effectiveness to different predators. *Formosan Entomologist* **32**: 41–57.

Chen SC, Chen ZC. 1997. The breeding of the firefly, *Luciola ficta*. *Bulletin of National Ilan Institute of Agriculture and Technology* **14**:25–32.

Chen Y-R, David T, Owens ACS, Wu C-H, Hsiao C-Y, Tang H-C, Zhong S, Yang E-C. 2018. The impact of artificial light on *Aquatica ficta* larvae transcriptome. *Formosan Entomologist* **38**:63–72 DOI: 10.6662/TESFE.2018007

Chen Y-R, Wei W-L, Tzeng DTW, Owens ACS, Tang H-C, WU C-S, Lin S-S, Zhong S, Yang E-C. 2021. Effects of artificial light at night (ALAN) on gene expression of *Aquatica ficta* firefly larvae. *Environmental Pollution* **281**:116944 DOI: 10.1016/j.envpol.2021.116944

Christensen TA, Carlson AD. 1981. Evidence for non-modulatory octopaminergic transmission mediating luminescence in larval fireflies. *Society for Neuroscience Abstracts* **7**:413.

Christensen TA, Carlson AD. 1982. The neurophysiology of larval firefly luminescence: direct activation through four bifurcating (DUM) neurons. *Journal of comparative physiology* **148**:503–514 DOI: 10.1007/BF00619788

Christensen TA, Sherman TG, McCaman RE, Carlson AD. 1983. Presence of octopamine in firefly photomotor neurons. *Neuroscience* **9**(1):183–189 DOI: 10.1016/0306-4522(83)90055-6

Cicero JM. 1982. The genus *Bicellonycha* in the United States with descriptions of a new species and subspecies (Coleoptera: Lampyridae, Photurinae). *The Coleopterists Bulletin* **36**(2): 270–278.

Cicero JM. 1994. Composite, haustellate mouthparts in netwinged beetle and firefly larvae (Coleoptera, Cantharoidea: Lycidae, Lampyridae). *Journal of Morphology* **219**(2):183–192 DOI 10.1002/jmor.1052190207

Copeland J. 1981. Effects of larval firefly extracts on molluscan cardiac activity. *Experientia* **37**:1271–1272. DOI: 10.1007/BF01948354

Costa C, Vanin SA, & Casari-Chen AS. 1988. *Larvas de Coleoptera do Brasil*. São Paulo: Museu de Zoologia da Universidade de São Paulo.

Cros A. 1924. *Pelania mauritanica* L. variations - moeurs - evolution. *Bulletin de la Société d'histoire naturelle de l'Afrique du Nord* **15**:10–52.

Davydova ED. 1968. A larva of the glow-worm *Lampyris orientalis* (Coleoptera, Lampyridae) (in Russian). *Zoologicheskii Zhurnal* **47**:1101–1103.

Day JC. 2011. Parasites, predators and defence of fireflies and glow-worms. In Day JC, ed. *Lampyrid: The Journal of Bioluminescent Beetle Research Volume 1*. Oxfordshire: Brazen Head Publishing, 70–102.

De Cock R, Faust L, Lewis S. 2014. Courtship and mating in *Phausis reticulata*: male flight behaviors, female glow displays, and male attraction to light traps. *Florida Entomologist* **97:**1290–1307. DOI:10.1653/024.097.0404

De Cock R, Matthysen E. 2001. Do glow-worm larvae (Coleoptera: Lampyridae) use warning coloration? *Ethology* **107**:1019–1033. DOI: 10.1046/j.1439-0310.2001.00746.x

De Cock R, Matthysen E. 2003. Glow-worm larvae bioluminescence (Coleoptera: Lampyridae) operates as an aposematic signal upon toads (*Bufo bufo*). *Behavioral Ecology* **14**(1):103–108. DOI: 10.1093/beheco/14.1.103

De Cock R. 2000. Rare, or simply overlooked? Practical notes for survey and monitoring of the small glow-worm *Phosphaenus hemipterus* (Coleoptera: Lampyridae). *Belgian Journal of Zoology* **130**:93–101.

De Cock R. 2004. Larval and adult emission spectra of bioluminescence in three European species of fireflies (Coleoptera: Lampyridae). *Photochemistry and Photobiology* **79**(4):339–342 DOI: 10.1562/2003-11-11-RA.1

De Cock R., Geisthardt M. 2007. Description of the adult female and larval stages of *Lampyris sardiniae* Geisthardt, 1987 (Coleoptera: Lampyridae). *Deutsche Entomologische Zeitschrift* **117(3)**:99–102.

De Cock R., Matthysen E. 1999. Aposematism and bioluminescence: experimental evidence from glow-worm larvae (Coleoptera: Lampyridae). *Evolutionary Ecology* **13**:619–639. DOI: 10.1023/A:1011090017949

De Cock R., Nepomuceno AH., Oliveira NG, Gomes J. 2015 *Fireflies and Glow-worms of Portugal - Guia Pirilampos de Portugal* . Vila Nova de Gaia: Águas e Parque Biológico de Gaia, Câmara Municipal de Vila Nova de Gaia.

De Maria M, Pellegrino J, Okabe K. 1967. Predatory activity of *Luciola cruciata* (Olivier, 1886) (Coleoptera: Lampyridae) larvae on newly-hatched *Biomphalaria glabrata*. *Mushi* **41(9):**121–122.

Dean MB. 1979. *The Natural History of Pterotus obscuripennis LeConte (Lampyridae, Coleoptera).* Arcata, California*:* Humboldt State University (Master of Arts Thesis).

Deheyn DD, Ballantyne, LA. **2009**. Optical characterization and redescription of the South Pacific firefly *Bourgeoisia hypocrita* Olivier (Coleoptera: Lampyridae: Luciolinae). *Zootaxa*, **2129**:47–62 DOI: 10.11646/zootaxa.2129.1.3

Domagala P, Ghiradella H. 1984. Structure and function of the terminal abdominal appendages (pygypodia) of photurid firefly larvae. *Biological Bulletin* **166**: 299–309. DOI: 10.2307/1541218

Dreisig H. 1974. Observations on the Luminescence of the larval glowworm, *Lampyris noctiluca* L. (Col. Lampyridae). *Insect Systematics & Evolution* **5**(2):103–109 DOI: 10.1163/187631274X00128

Fabre H. 1913. The glow-worm. The first user of anaesthetics. *The Century Magazine* **87**:105–112.

Fabre JH. 1924. *The glow-worm and other beetles* (translated by Alexander Teixeira de Mattos) New York: Dodd, Mead And Company.

Fairmaire L. 1900. Description d’une Luciole nouvelle de Madagascar et de sa larve [Col.]. *Bulletin de la Société Entomologique de France* **1900**:361–363.

Fallon TR, Lower SE, Chang CH, Bessho-Uehara M, Martin GJ, Bewick AJ, Behringer M, Debat HJ, Wong I, Day JC, Suvorov A, Silva CJ, Stanger-Hall KF, Hall DW, Schmitz

RJ, Nelson DR, Lewis SM, Shigenobu S, Bybee SM, Larracuente AM, Oba Y, Weng J-K. 2018. Firefly genomes illuminate parallel origins of bioluminescence in beetles. *eLife* **2018;7**:e36495 DOI: 10.7554/eLife.36495

Fang L, Yang J-W, Wang J-L, Zhu J-Q, Fu X-H. 2013. Preliminary investigation of predation of the snail *Bradybaena ravida* by larvae of the firefly *Pyrocoelia pectoralis*. *Chinese Journal of Applied Entomology* **50**(1):197–202.

Faust L, Faust H. 2014. The occurrence and behaviors of North American fireflies (Coleoptera: Lampyridae) on milkweed, *Ascleplas syriaca* L. *Coleopterists Bulletin* **68**(2):283–291. DOI: 10.1649/0010-065X-68.2.283

Faust L, Forrest TG. 2017. Bringing light to the lives of the shadow ghosts, *Phausis inaccensa*. *American Entomologist* **63**:177–189 DOI doi.org/10.1093/ae/tmx027

Faust L. 2012. Fireflies in the snow: Observations on two early-season arboreal fireflies *Ellychnia corrusca* and *Pyractomena borealis*. In Kirton LG, Day JC, Lim GT. (ed). *Lampyrid: Volume 2 2012: The Journal of Bioluminescent Beetle Research (Lampyrid Journal)*. Oxfordshire: Brazen Head Publishing, 48–71.

Faust LF. 2010. Natural history and flash repertoire of the synchronous firefly Photinus carolinus (Coleoptera: Lampyridae) in the Great Smoky Mountains National Park. *Florida Entomologist* **93**(2):208–217 DOI: 10.1653/024.093.0210

Faust LF. 2017. *Fireflies, Glow-worms, and Lightning Bugs. Identification and Natural History of the Fireflies of the Eastern and Central United States and Canada*. Athens: University of Georgia Press.

Fletcher M. 1919 Second hundred notes on Indian insects. 135. Larva of *Luciola gorhami*. *Bulletin of the Agricultural Research Institute,* Pusa, New Delhi **89(2)**:28–29.

Foo K, Seelan J, Dawood M. 2017. Microfungi associated with *Pteroptyx bearni* (Coleoptera: Lampyridae) eggs and larvae from Kawang River, Sabah (Northern Borneo). *Insects* **2017**, 8, 66 DOI: 10.3390/insects8030066

Forsyth DJ, Peterle TJ, Bandy LW. 1983. Persistence and transfer of 36Cl- DDT in the soil and biota of an old-field ecosystem: a six-year balance study. *Ecology* **64**(6):1620–1636 DOI: 10.2307/1937515

Fu X, Ballantyne L, Lambkin C. 2012a. *Emeia* gen. nov., a new genus of Luciolinae fireflies from China (Coleoptera: Lampyridae) with an unusual trilobite-like larva, and a redescription of the genus *Curtos* Motsch. *Zootaxa* **3403**:1–53 DOI: 10.11646/zootaxa.3403.1.1

Fu X, Ballantyne L, Lambkin C. 2012b. The external larval morphology of aquatic and terrestrial Luciolinae fireflies (Coleoptera: Lampyridae). *Zootaxa* **3405**:1–34. DOI: 10.11646/zootaxa.3405.1.1

Fu X, Ballantyne L. 2006. *Luciola leii* sp. nov., a new species of aquatic firefly (Coleoptera: Lampyridae: Luciolinae) from mainland China. *The Canadian Entomologist,* **138(3)**: 339–347 Doi:10.4039/n05–102.

Fu X, Meyer-Rochow VB, Tyler J, Suzuki H, De Cock R. 2009. Structure and function of the eversible organs of several genera of larval firefly (Coleoptera: Lampyridae). *Chemoecology* **19**:155–168

Fu X, Meyer-Rochow VB. 2012. An investigation into the morphological and behavioral adaptations of the aquatic larvae of *Aquatica leii* (Coleoptera: Lampyridae) to prey upon freshwater snails that serve as intermediate hosts for the liver fluke. *Biological Control* **62**(3):127–134. DOI 10.1016/j.biocontrol.2011.12.007

Fu X, Meyer-Rochow VB. 2013. Larvae of the firefly *Pyrocoelia pectoralis* (Coleoptera: Lampyridae) as possible biological agents to control the land snail *Bradybaena ravida*. *Biological Control* **65**(2):176–183. DOI 10.1016/j.biocontrol.2013.02.005

Fu X, Meyer-Rochow VB. 2021. Selection and validation of suitable reference genes for RT-qPCR analysis in the rare aquatic firefly *Aquatica leii* (Coleoptera: Lampyridae). *Insects* **12**(4):359 DOI: 10.3390/insects12040359

Fu X, Vencl F, Nobuyoshi O, Meyer-Rochow V, Lei CL, Zhang Z. 2007. Structure and function of the eversible glands of the aquatic firefly *Luciola leii* (Coleoptera: Lampyridae). *Chemoecology* **17**:117–124 DOI: 0.1007/s00049-007-0370-3

Fu X, Wang Y, Lei C, Ohba, N. 2005b. The swimming behavior of the aquatic larvae of the firefly *Luciola substriata* (Coleoptera: Lampyridae). *The Coleopterists Bulletin* **59**(4):501–505. DOI: https://doi.org/10.1649/830.1

Fu XH, Ballantyne L.A. Lambkin C. 2010. *Aquatica* gen. nov. from mainland China with a description of *Aquatica wuhana* sp. nov. (Coleoptera: Lampyridae: Luciolinae). *Zootaxa* **2530**:1–18 DOI: 10.11646/zootaxa.2530.1.1

Fu XH, Ballantyne LA. 2008. Taxonomy and behaviour of lucioline fireflies (Coleoptera: Lampyridae: Luciolinae) with redefinition and new species of *Pygoluciola* Wittmer from mainland China and review of *Luciola* LaPorte. *Zootaxa*, **1733**:1–44 DOI: 10.11646/zootaxa.1733.1.1

Fu X-H, Ohba N, Lei C-L. 2004. Morphological and biological observations on aquatic firefly *Luciola substriata* (Gorham) (Coleoptera: Lampyridae) in China. *Acta Entomologica Sinica* **47**(3):372–378.

Fu XH, Ohba N, Vencl FV, Lei CL. 2005a. Structure, behavior, and the life cycle of an aquatic firefly, *Luciola substriata*, in China. *The Canadian Entomologist* **137**:83–90 DOI: 10.4039/n04-022

Fu XH, Ohba N, Vencl FV, Lei CL. 2006a. Life cycle and behaviour of the aquatic firefly *Luciola leii* (Coleoptera: Lampyridae) from Mainland China. *The Canadian Entomologist* **138**:860–870. DOI: 10.4039/n05-093

Fu XH, Ohba N, Zhang Y, Lei CL. 2006b. A rearing apparatus and diet for the aquatic firefly, *Luciola leii n sp* (Coleoptera: Lampyridae). *The Canadian Entomologist* **138**(3):399–406. DOI: 10.4039/n05-029

Fu X-H, Wang YY, Lei CL. 2005. Adaptive external morphology and swimming behavior in the aquatic firefly, *Luciola substriata*. *Kunchong Zhishi* **42**(4):419–423.

Fu X-H. 2009. Defensive behavior in firefly *Pyrocoelia pectoralis* (Coleoptera: Lampyridae): reflex bleeding and reaction with eversible organs. *Acta Entomologica Sinica* **52**(7):783–790.

Fu, XH & Ballantyne, LA. 2009. Larval respiration system and evolution in aquatic fireflies (Coleoptera: Lampyridae: Luciolinae). in Meyer-Rochow VB, ed. *Bioluminescence in Focus: A Collection of Illuminating Essays.* Thiruvananthapuram: Research Signpost, 243–254.

Ganguly G, Ghosh AK. 1982. Feeding behaviour and digestive enzymes of luminous larvae of *Luciola gorhami* Ritz (Lampyridae: Coleoptera). *Journal of the Zoological Society of India* **34:**1–6.

Gardner JCM. 1946. Larvae of Cantharoidea (Coleoptera). *Indian Journal of Entomology,* **8(1)**:121–129.

Geisthardt M. 1979. Skelet und muskulatur des thorax der larven und imagines von *Lamprohiza splendidula* (L.) unter Berucksichtigung der larve und der weiblichen Imago von *Lampyris*

noctiluca (L.) (Coleoptera: Lampyridae). *Zoologische Jahrbucher. Abteilung fur Anatomie und Ontogenie der Tiere* **101**:472–536.

Geisthardt M. 2007. A new polytypic *Lampyris* from Italy: *Lampyris vesuvius vesuvius* sp. n. and *Lampyris vesuvius insularis* ssp. n. (Coleoptera: Lampyridae). In Nardi G, Vomero V. eds. *Artropodi del parco nazionale del Vesuvio: ricerche preliminari.* Verona, Cierre, 185–190.

Gentry E. 2003. On sexual selection in Florida's *Pyractomena borealis* (Coleoptera: Lampyridae). *The Florida Entomologist* **86**(2):114–123 DOI 10.1653/0015-4040(2003)086[0114:OSSIFP]2.0.CO;2

Ghigi A. 1901. La larva della *Luciola italica*. *Bullettino della Società Entomologica Italiana* **33**:183–189.

Gorgadze O, Tskhadaia E. 1995. Investigation of the biology of the big firefly (*Lampyris noctiluca*) in Eastern Georgia with a view of artificial reproduction. *Bulletin of the Georgian Academy of Sciences* **152**(1): 171–174

Gorgadze O. 1998. Population of the big firefly (*Lampyris noctiluca*) and some of its biological characteristics. *Bulletin of the Georgian Academy of Sciences* **158**(3):507–508

Gotou M, Sekine M, Kanao M, Miyamoto K, Higuchi T, Imai T, Ukita M. 2005. Survey on the effectiveness of riverbank protection works for firefly. *Doboku Gakkai Ronbunshu* **2005**(804): 11–22 DOI https://doi.org/10.2208/jscej.2005.804_11

Gunn P, Gunn B. 2013. Lunar effects on the bioluminescent activity of the glow-worm *Lampyris noctiluca* and its larvae. In: Day JC, ed. Lampyrid Volume 3 (2013) Oxfordshire: Brazen Head Publishing, 1–22.

Haddon K. 1915. On the methods of feeding and the mouthparts of the larva of the glow-worm (*Lampyris noctiluca*). *Proceedings of the Zoological Society of London* **1915**(1-2):77–82.

Hadj-Mohammadi MR, Chaichi M.J. 1996. Separation, identification and determination of luciferin in the Iranian firefly, *Lampyris turkestanicus* by HPLC and spectroscopic methods. *Photochemistry and Photobiology* **64**(5):821–822. doi:10.1111/j.1751-1097.1996.tb01841.x

Hanneda Y. 1977. Report on experimental process of cultivation of Japanese fireflies *Luciola cruciata* and *L. lateralis*. *Annual Report of Yokosuka City Museum* **23**:23–26.

Hara S. 1962. Larvae of *Luciola cruciata* and *L. lateralis* (Coleoptera). *Kontyu Tokyo* **30**:230–235.

Harvey EN, Hall RT. 1929. Will the adult firefly luminesce if its larval organs are entirely removed? *Science* **69(1783)**:253–254 DOI: 10.1126/science.69.1783.253

Hasama B. 1942a. Uber die Biolumineszenz der *Luciola lateralis* im zytologischen Bild sowie im Potentialbild ihres Leuchtorgans. *Cytologia* **12**(4): 366–377 DOI 10.1508/cytologia.12.366

Hasama B. 1942b. Über die Biolumineszenz der Larve von *Luciola cruciata* sowie von *Pyrocoelia rufa* im Aktionsstrombild und im histologischen Bild ihres Leuchtorgans. *Cytologia* **12**(4): 378–388. DOI 10.1508/cytologia.12.378

Hashmi AA, Asghar MA, Hamed M. 1986. Scanning of taxonomic characters in coleopterous larvae. **Pakistan Journal of Zoology** *18*(2):139–143.

Hastings, JW, Buck J. 1956. The firefly pseudoflash in relation to photogenic control. *The Biological Bulletin* **111(1)**:101–113 DOI: 10.2307/1539187

Hatano T, Kato T. 1963. Laboratory studies on the effects of agricultural chemicals on the first stage larvae of a firefly, *Luciola lateralis* Motschulsky. *Science Bulletin of the Faculty of Agriculture Kyushu University Fukuoka* **20**:265–269.

Hayashi N. 1991. Lampyrid beetles in Kawasaki and their larvae. Kawasaki Shizen-Kankyô *Chôsa Hôkoku* **2:**117–127.

Hess WN. 1920. Notes on the biology of some common Lampyridae. *The Biological Bulletin,* **38(2)**:39–76 DOI: 10.2307/1536232

Hess WN. 1922. Origin and development of the light organs of *Photuris pennsylvanica* De Geer. *Journal of Morphology* **36(2):**245–277.

Ho J.Z, Chiang P.H, Wu CH, Yang PS. 2010. Life cycle of the aquatic firefly *Luciola ficta* (Coleoptera: Lampyridae). *Journal of Asia-Pacific Entomology*, **13(3):**189–196.

Ho JZ, Chiang CP, Yang PS. 2006. A new rearing method for an aquatic firefly, *Luciola ficta* (Coleoptera: Lampyridae). *Formosan Entomologist* **26**:305–312.

Ho JZ, Chiang PH. 1997. Two firefly species with aquatic larvae in Taiwan. *Nature Conservation Quarterly* **17**:42–46.

Ho JZ, Chiang PH. 2002. *Shadows of firefly glowing on the water—the conservation and recovery of aquatic fireflies.* Nantou, Chichi: Taiwan Endemic Species Research Institute.

Ho JZ, Chu CS, Chu CC. 1998. A discovery of the aquatic larvae of *Luciola substriata*. *Nature Conservation Quartely* **22**:47–51.

Ho J-Z, Fang H-T, Yang P-S. 2014. Breeding apparatus of the terrestrial firefly, *Luciola cerata* (Coleoptera: Lampyridae). *Formosan Entomologist* **33**:281–290 DOI: 10.6661/TESFE.2013020

Ho JZ, Huang SW. 2003. Effects of temperature and egg size on egg duration, hatching rate, and starvation tolerance of first instar larvae of the firefly *Pyrocoelia analis*. *Formosan Entomologist* **24:**305–312 DOI: 10.6661/TESFE.2003028

Ho JZ, Jong RF. 1997. *Pristolycus kanoi* in crisis: morphology and behavior. *Nature Conservation Quartely* **18:**26–31.

Ho JZ, Su TH, Huang SW. 2003. Rearing methods and life cycle of *Pyrocoelia analis* (Coleoptera: Lampyridae). *BioFormosa* **38:**79–87.

Ho JZ, Su TH. 2000. Morphology and functions of the prolegs of the firefly *Luciola gorhami* Ritsema larvae (Coleoptera: Lampyridae). *Endemic Species Research* **2:**54–60.

Ho JZ, Fung HT, Hu JH, Yang PS. 2014. Ants as a diet for the life cycle of the terrestrial firefly *Luciola ceraeta* (Coleoptera: Lampyridae). *2014 International Firefly Symposium. Univ. Florida, Gainesville, Florida* 11–15. Available at https://conference.ifas.ufl.edu/firefly/

Ho JZ. 2002. *Larval morphology of twenty-one species and bionomics of fireflies (Coleoptera: Lampyridae) in Taiwan.* Doctoral dissertation in the Department of Entomology, National Chung Hsing University Taiwan.

Ho J-Z. 2004. Occurred fluctuation, distribution and habitat characters of the firefly, *Pyrocoelia analis*. *Formosan Entomologist* **24**(2):117–128 DOI: 10.6661/TESFE.2004011

Horne J, Horne A. 2017. Larval development rates in the glow-worm *Lampyris noctiluca* (L.). In: Day JC, ed. *Lampyrid: Volume 4 (2017): The Journal of Bioluminescent Beetle Research (Lampyrid Journal)*. Oxfordshire: Brazen Head Publishing, 55–58.

Hutson JC, Austin GD. 1924. Notes on the habits and life-history of the Indian glow-worm, an enemy of the African or Kalutara snail. *Ceylon Departament of Agriculture Bulletin* **69**, 16p.

Imms AD. 1933. Scientific results of the Cambridge expedition to the east African Lakes, 1930-1.-10. On some aquatic Coleopterous larvae. *Journal of the Linnean Society of London, Zoology* **38**:301–307 DOI 10.1111/j.1096-3642.1933.tb00061.x

Imuta N, Nakamura K, Hirata H. 1994. Feeding experiments of larval firefly *Luciola picticollis* with artificial pellets. *Memoirs of Faculty of Fisheries Kagoshima University* **43**:61–67.

Ineichen S. 2004. Zur Raumnutzung von Larven, Weibchen und Männchen des Grossen Glühwürmchens Lampyris noctiluca (Coleoptera, Lampyridae). *Mitteilungen der Entomologischen Gesellschaft Basel* **53(4)**:111–122.

Jaikla S, Thancharoen A, Pinkaew N. 2020. Biology and rearing technique for the mangrove firefly, *Pteroptyx valida* (Coleoptera: Lampyridae) Olivier, with discussion of additional instar in female. *Journal of Asia-Pacific Biodiversity* **13**(3):367–371 DOI: 10.1016/j.japb.2020.05.002

Janisova K, Bocakova M. 2013. Revision of the subfamily Ototretinae (Coleoptera: Lampyridae). *Zoologischer Anzeiger* **252**:1–19 DOI: 10.1016/j.jcz.2012.01.001

Jeng ML, & Yang PS, Satô M, Lai J, Chang JC. 1998. The genus *Curtos* (Coleoptera, Lampyridae, Luciolinae) of Taiwan and Japan. *Japanese Journal of Systematic Entomology* **4 (2)**:331–347.

Jeng M-L, Suzuki Y, Chang C-Y, Chen, T-R. 2021. Do holometabolous insects molt spontaneously after adulthood? An exceptional case report in fireflies (Coleoptera: Lampyridae), with discussion of its inferred endocrine regulation especially in relation to neoteny. *Arthropod Structure & Development* **61**:101013 DOI: 10.1016/j.asd.2020.101013

Jeng ML., Lai J. & Yang PS. 2003. Lampyridae: A synopsis of aquatic fireflies with description of a new species. In: Jäch, MA, Ji L. eds. *Water Beetles of China Vol. III.* Wien: Zoologisch-Botanische Gesellschaft in Österreich, Wiener Coleopterologenverein, 539–562.

Kakehashi K, Kuranishi RB, Kamata N. 2014. Estimation of dispersal ability responding to environmental conditions: larval dispersal of the flightless firefly, *Luciola parvula* (Coleoptera: Lampyridae). *Ecological research* **29**(5):779–787 DOI: 10.1007/s11284-014-1156-z

Kakehashi K, Kuranishi RB, Kamata N. 2013. Environmental factors affecting the spatial distribution and activity of firefly larvae *Luciola parvula* (Coleoptera: Lampyridae: Luciolinae): high activity under rich soil moisture. *Japanese Journal of Conservation Ecology* **18**:45–54.

Kanda S. 1934. Study of firefly (1): Life cycle of *Luciola cruciata*. *Kontyu* **8**:67–73.

Kanjana I, Sriboonlert A, E-Kobon T, Thancharoen A, Chumnanpuen P. 2017. Comparison of the pronotum integument of firefly larvae; *Lamprigera* sp., *Pyrocoelia* sp., *Pteroptyx* sp., and *Sclerotia aquatilis* (Coleoptera: Lampyridae). *Research 4.0 Innovation and Development SSRU's 80th Anniversary:* 49–57.

Katsuno S. 1963. *Luciola cruciata* of Tatsuno Town, Nagano Prefecture, and its cultivation. *Miscellaneous Report of the Yokosuka City Museum* **9**:1–6.

Katsuno Sakyo. 1968. Breeding fireflies by artificial incubation in Nagano. *Insects* **6**:13–17

Kaufmann T. 1965. Ecological and Biological Studies on the West African firefly *Luciola discicollis* (Coleoptera: Lampyridae). *Annals of the Entomological Society of America* **58(4)**:414–426.

Kawashima I, Nagai K, Horiuchi Y, Yagishita Y, Takanashi S. 2016. Lampyrid beetles in the Ikuta Ryokuchi Park, Kawasaki City (2nd report), with description of 1st instar larva of *Pristolycus sagulatus* Gorham, 1883 (Coleoptera, Lampyridae). *Kawasaki Municipal Science Museum* **26**:11–16.

Kawashima I, Satou F. 2004. The lampyrid genus *Stenocladius* (Coleoptera, Lampyridae) from the Okinawa Islands, Middle Ryukyus, Southwest Japan, with descriptions of two new local populations. *Elytra* **32**:389–403.

Kawashima I. 2017a. Larval morphology of the lampyrine species, *Lucidina accensa* Gorham (Coleoptera: Lampyridae: Lampyrinae) from Honshû, Japan. *Japanese Journal of Systematic Entomology* **23(1)**:129–134.

Kawashima I. 2017b. Larval morphology of the cyphonocerine species, *Cyphonocerus okinawanus* Nakane (Coleoptera, Lampyridae, Cyphonocerinae) from Amami and Okinawa islands, the Middle Ryûkyûs, southwestern Japan. *Japanese Journal of Systematic Entomology* **23(2):**239–246.

Kawashima I. 2018. Larval Morphology of two Lucioline Species, *Curtos costipennis* (Gorham) and *C. okinawanus* Matsumura (Coleoptera: Lampyridae: Luciolinae) from the Ryukyu Islands, Southwestern Japan. *Japanese Journal of Systematic Entomology* **24**(1):127–137.

Kawashima I. 2019a. Redescription of larva of the Lucioline species, *Luciola filiformis yayeyamana* Matsumura (Coleoptera, Lampyridae, Luciolinae) from the Yaeyama Islands, SW Ryûkyûs, Japan. *Japanese Journal of Systematic Entomology* **25(1)**:115–121.

Kawashima I. 2019b. Supposed larva of *Lucidina natsumiae* Chujo & Sato (Coleoptera, Lampyridae, Lampyrinae) from the Yaeyama Islands, SW Ryukyus. *Japanese Journal of Systematic Entomology* **25**:107–113.

Kawashima I. 2020a. Redescription of larva of *Pristolycus sagulatus sagulatus* Gorham (Coleoptera: Lampyridae: Luciolinae) from the Kanto Region, Honshu, Japan. *Japanese Journal of Systematic Entomology* **26**(1):165–172.

Kawashima I. 2020b. Taxonomic review of '*Luciola tsushimana* Nakane' (Coleoptera: Lampyridae: Luciolinae) from Tsushima Is., Japan, with description of the larva. *Japanese Journal of Systematic Entomology* **26**(2):261–274.

Kawashima l, Takai Y. 2004. Immature stages and adult female of the Lampyrinae species, *Lucidina okadai* Nakane et Ohbayashi, 1949 (Coleoptera, Lampyridae, Lampyrinae) from Gifu, Central Japan. *Elytra* **32(1)**:153–170.

Keiper RR, Solomon LM. 1972. Ecology and yearly cycle of the firefly *Photuris pennsylvanica* (Coleoptera: Lampyridae). *Journal of the New York Entomological Society* **80**:43–47.

Kiichiro M. 1961. Hotaru no kenkyu (A study of fireflies). Published by the author. Moriyama, Shiga Prefecture, Japan.

Kim HG, Kwon YJ, Suh SJ. 2008. Bionomical characteristics of *Luciola lateralis* (Coleoptera: Lampyridae) in mass breeding. *Journal of Life Science* **18**(12):1728–1732. DOI 10.5352/JLS.2008.18.12.1728

King HS. 1880. Life history of *Pleotomus pallens* LeC. *Psyche* **3(72)**:51–53.

Kok PJ, van Doorn L, Dezfoulian R. 2019. Predation by non-bioluminescent firefly larvae on a tepui-summit endemic toad. *Current Biology*, **29**(22), R1170-R1171 Doi: 10.1016/j.cub.2019.10.001

Konda A, Tanaka F. 1989. An experimental study of predation by the larvae of the firefly, *Luciola lateralis* Motschulsky (Coleoptera: Lampyridae) on the apple snail, *Pomacea canaliculata* Lamarck (Mesogastropoda: Pilidae). *Japanese Journal of Applied Entomology and Zoology* **33**:211–216 DOI: 10.1303/jjaez.33.211

Kondo H, Yagi T. 2007. Effect of flood on population dyanamics [dynamics] of Japanese aquatic firefly *Luciola cruciata* and fresh water snail *Semisulcospira* sp. in Gunke-River, Hyogo Prefecture. *Humans and Nature* **17**:67–72 DOI: 10.24713/hitotoshizen.17.0_67.

Korschefsky R. 1951. Bestimmungstabelle der bekanntesten deutschen Lyciden-, Lampyriden- und Drilidenlarven (Coleoptera). *Beiträge zur Entomologie* **1(1)**:60–64.

Kumode M, Tanaka K, Yuma M. 1999. Estimation of the peak flight season of adult Genji-firefly, *Luciola cruciata* (Coleoptera). *Ecology and Civil Engineering* **2**(2):205–210. DOI: 10.3825/ece.2.205

Kusui Y. 1979. Record of fireflies in the Miyako Islands, Okinawa, with the observation on predation of the land snail (*Aegista oculus*) by a larva of firefly (*Lychnuris* sp.). *Nanki Seibutu* **21**(1):34.

Kyuka T, Shimizu Y, Osawa T, Ishida Y, Sasaki H, Inamoto Y, Mitsuhashi H. 2010. New attempt in restoration to create habitat of the firefly larva (*Luciola cruciata*) and the marsh snail (*Semisulcospira libertine*) using spur dike in concrete river - construction method and its advantage. *Humans and Nature* **21**:159–165.

LaBella DM, Lloyd JE. 1991. Lampyridae (Cantharoidea). In: Stehr FW, ed. *Immature Insects. Vol. 2.* Dubuque: Kendall Hunt Publishing Co, 427–428.

Lanuza-Garay A, Santos-Murgas A, Barría EA, Hernández GC, Osorio-Arenas MA. 2021. Depredación de la "babosa" *Veronicella cubensis* Pfeiffer (Mollusca: Gastropoda: Veronicellidae), por la larva de *Cratomorphus signativentris* Olivier 1895 (Coleoptera: Lampyridae) en Panamá. *Tecnociencia* **23**(1): 339–350 DOI: 10.48204/j.tecno.v23n1a18

Lee DW, Boo KS. 1991. Studies on the light organ of the firefly, *Luciola lateralis* Motschulsky. *Korean Journal of Applied Entomology* **30**(1):29–36

Lee KY, Ahn KS, Kang HJ, Park SK, Kim JG. 2003. Effects of temperature on reproduction and development of firefly, *Luciola lateralis* (Coleoptera: Lampyridae) *Korean Journal of Applied Entomology* **42**(3):217–223.

Lee KY, Kim YH, Lee JW, Song MK, Nam SH. 2008. Toxicity of firefly, *Luciola lateralis* (Coleoptera: Lampyridae) to commercially registered insecticides and fertilizers. *Korean Journal of Applied Entomology* **47**(3):265–272. DOI: 10.5656/KSAE.2008.47.3.265

Lehtonen TK, Babic NL, Piepponen T, Valkeeniemi O, Borshagovski, A-M, Kaitala A. 2021. High road mortality during female-biased larval dispersal in an iconic beetle. *Behavioral Ecology and Sociobiology* **75(1)**:26 DOI: 10.107/s00265-020-02962-6

Lequet A, Faucheux M. 2015. Un cas de prothételie chez le ver luisant, *Lampyris noctiluca* (Linnaeus, 1967) (Coleoptera: Lampyridae). *Bulletin de la Société des Sciences Naturelles de l'Ouest de la France* **37**(4):205–231.

Lequet A, Faucheux M. 2016. Rôles et morphologie des tentacules pygopdiens de la larve de ver luisant *Lampyris noctiluca* (Linnaeus, 1767) (Coleoptera: Elateroidea: Lampyridae). *Bulletin de la Société des Sciences Naturelles de l'Ouest de la France* **38**(3):130–139.

Lewis SM, Wong CH, Owens ACS, Fallon C, Jepsen S, Thanchareon A, Wu C, De Cock R, Novák M, López-Palafox T, Khoo V, Reed MJ. 2020. A Global Perspective on firefly extinction threats. *BioScience* **70**(2):157–167 DOI doi.org/10.1093/biosci/biz157

Lheritier G. 1955. Observations sur le comportment de *Pelania mauritanica* L. *Societe des Sciences Naturelles et Physiques de Maroc* **35**:223–233.

Li X-Y, Xie M, Dong P-X, Liang X-C. 2008. Morphology of *Pyrocoelia pygidialis* Pic (Coleoptera: Lampyridae) with notes on its biology. *Entomotaxonomia* **30**(4):300–308.

Liew T, Schilthuizen M. 2014. Association between shell morphology of micro-land snails (genus *Plectostoma*) and their predator's predatory behaviour. *PeerJ* **2**:e329 DOI 10.7717/peerj.329

Lloyd JE, Wing SR, Hongtrakul T. 1989. Ecology, flashes and behavior of congregating Thai fireflies. *Biotropica* **21**(4):373–376 DOI: 10.2307/2388290

Lloyd JE. 1973a. Firefly Parasites and Predators. *The Coleopterists Bulletin* **27**:91–106.

Lloyd JE. 1973b. Firefly Inhabitant of coastal reefs in New Guinea (Coleoptera: Lampyridae). *Biotropica* **5**(3):168–174 DOI: 10.2307/2989809

Lloyd JE. 1973c. Fireflies, commonplace beetles and larvae by day, but tiny flashing lanterns on summer evenings. *Animals* **15**:220–225.

Lloyd JE. 2006. Stray light, fireflies, and fireflyers. In Rich C, Longcore T, eds. *Ecological consequences of artificial night lighting.* Washington, D.C.: Island Press, 345–364.

Lloyd, JE. 2018. *A Naturalist's long walk among shadows of North American Photuris: patterns, outlines, silhouettes... Echoes.* Gainesville: Bridgen Press.

Loomboot S, Jamornmarn S, Chankaew K, Chongrattanameteekul W. 2007. The biology and rearing of firefly *Pteroptyx malaccae* Gorham. *Environment and Natural Resources Journal* **5(1)**:35–43.

Lucas H. 1904. Description d'une larve géante appartenant a la famille des Lampyrides. In Pavie A. ed. *Mission Pavie Indo-Chine I879-I895. Études Diverses III. Recherches sur L'Histoire Naturelle de L'Indo-Chine Orientale*. Paris, Ernest Leroux, 104–105.

Maas U, Sehn E, Harris JR, Dorn A. 2001. Ergastoplasmic paracrystalline inclusion bodies in the adipose gonadal envelope and fat body of the glow worm, *Lampyris noctiluca* (Insecta, Coleoptera). *Micron* **32**(2):129–140. DOI: 10.1016/s0968-4328(00)00002-0

Madruga-Rios O, Branham MA. 2020. Description of life cycle and preimaginal stages of *Alecton discoidalis* Laporte, 1833 (Coleoptera: Lampyridae) under laboratory conditions. *Zootaxa* **4816**(1):81–91 DOI: 10.11646/zootaxa.4816.1.4

Madruga-Rios O, Hernández-Quinta M. 2010. Larval feeding habits of the Cuban endemic Firefly *Alecton discoidalis* Laporte (Coleoptera: Lampyridae). *Psyche* **2010**, ID 149879 DOI: 10.1155/2010/149879

Madruga-Rios O. 2018. Seleccion alimentaria de las larvas de las luciernaga cubana *Alecton discoidallis Boletín de la Sociedad Entomológica Aragonesa (S.E.A.)* **62**:321–322.

Majka CG, MacIvor JS. 2009. The European lesser glow worm, *Phosphaenus hemipterus* (Goeze), in North America (Coleoptera, Lampyridae). *ZooKeys* **29**:35–47. DOI: 10.3897/zookeys.29.279

Margry CJPJ. 2013. Escargot met uitjes? De glimworm *Lampyris noctiluca* (Linnaeus, 1767) (Coleoptera, Lampyridae) als gulzige slakkendoder. *Spirula Correspondentieblad van de Nederlandse Malacologische Vereniging* **392**:85–87.

Matsuda M, Oba Y, Konishi T, Oba Y. 2010. Survey on the larval habitats of terrestrial firefly, *Luciola parvula* in the campus of Nagoya University. *Bulletin of the Nagoya University Museum* **26**:153–163. DOI 10.18999/bulnum.026.14

Mbugua SW, Wong CH, Ratnayeke S. 2020. Effects of artificial light on the larvae of the firefly *Lamprigera* sp. in an urban city park, Peninsular Malaysia. *Journal of Asia-Pacific Entomology* **23**(1):82–85. DOI: 10.1016/j.aspen.2019.10.005

McDermott FA. 1954. The larva of *Micronaspis floridana* Green. *The Coleopterists Bulletin* **8(3/4)**:59–62.

McDermott FA. 1960. Fireflies of the Genus *Pyractonema* (Coleoptera: Lampyridae). *Proceedings of the United States National Museum* **112**(3433):133–157 DOI https://doi.org/10.5479/si.00963801.112-3433.133

McLean M, Buck J, Hanson F. 1972. Culture and larval behavior of photurid fireflies. *The American Midland Naturalist* **87**(1):133–145 DOI: 10.2307/2423887

Mehta DR. 1932. Fauna of Lahore 3. Preliminary notes on the life history of the firefly *Luciola gorhami* Rits., and cytology of the light organs. *Bulletin of the Department of Zooogy, Panjab University* **1**:107–118.

Mei Z-L, Cao C-Q, Tong C, Liu F-Q, Xu D-Y. 2020. Effects of different temperatures on the hatching of eggs and development of newly hatched larvae of *Emeia pseudosauteri*. *Huanjing Kunchong Xuebao* **42**(2):306–310.

Meinert F. 1886. Gjennemborede Kindbakker hos Lampyris- og Drilus-Larverne. *Entomologisk Tidskrift* **7:**194–196.

Minami K. 1966. *Hotaru no Kenkyu (A study of fireflies).* Published by author. Moriyama, Shiga Prefecture, Japan. 321p

Mobilim V, Dawood MM. 2020. Solitary fireflies of Kangkawat Research Station, Imbak Canyon, Sabah. *Journal of Tropical Biology and Conservation* **17**:131–147.

Moriya S, Yamauchi T, Nakagoshi N. 2006. Climbing behavior of mature larvae of Genji firefly, *Luciola cruciata* in Kure City, Japan (Coleoptera: Lampyridae). *Japanese Journal of Entomology New Series* **9**(3):59–68 DOI: 10.20848/kontyu.9.3_59

Moriya S, Yamauchi T, Nakagoshi N. 2007. Weight of the climbing larva of the firefly *Luciola cruciata* (Coleoptera: Lampyridae) and its relationship to size, weight, and sex of the adult. *The Entomological Review of Japan* **62(1):**127–134.

Moriya S, Yamauchi T, Nakagoshi N. 2009a. The pupal period of the firefly, *Luciola cruciata* (Coleoptera: Lampyridae) is decided depending on the weight of the climbing larva and temperature. *Humans and Nature* **20**:67–71.

Moriya S, Yamauchi T, Nakagoshi N. 2009b. Sex ratios in the Japanese firefly, *Luciola cruciata* (Coleoptera: Lampyridae) at emergence. *Japanese Journal of Limnology* **69**: 255–258 DOI: 10.3739/rikusui.69.255

Murphy F, Moiseff A. 2019. Anatomy of the stemmata in the *Photuris* firefly larva. *Journal of Comparative Physiology A* **205**:151–161 DOI: 10.1007/s00359-018-01312-2

Murphy F, Moiseff A. 2020. Ambient illumination influence on *Photuris* firefly larval surface movements is not mediated by the stemmata. *Journal of Insect Behavior* **33**:30–37 DOI: 10.1007/s10905-020-09743-z

Nada B, Ballantyne LA, Jusoh WFA. 2021. Description of the larva of a firefly species, *Pygoluciola dunguna* Nada (Coleoptera: Lampyridae). *Zootaxa* **4920**(4):528–542 DOI: 10.11646/zootaxa.4920.4.4

Naisse J. 1969 Role des neurohormones dans la différenciation sexuelle de Lampyris noctiluca. *Journal of Insect Physiology* **15**(5):877–878 DOI 10.1016/0022-1910(69)90128-0

Nakane T, Ohba N. 1981. *The observation and breeding of fireflies*. Tokyo: New Science Press.

Nathanson JA, Hunnicutt EJ. 1979. Neural control of light emission in *Photuris* larvae: identification of octopaminesensitive adenylate cyclase. *Journal of Experimental Zoology* **208**:255–262 DOI: 10.1002/jez.1402080213.

Natsumeda T, Matsuda T, Yuma M. 2013. Evaluation of habitat factors affecting aquatic fireflies in valley-bottom paddy fields in the northeast district of Chiba Prefecture, Japan. Japanese *Journal of Conservation Ecology* **18**(1):91–99 DOI: 10.18960/hozen.18.1_91

Newport G. 1857. On the natural history of the glow-worm (*Lampyris noctiluca*). *Proceedings of the Linnean Society of London* **69**:40–71.

Nishijima S, Yasuoka T, Maeto K. 2010. Survival and growth of *Luciola parvula* larvae fed land snails, earthworms, or wood lice (Coleoptera: Lampyridae). *Japanese Journal of Entomology* **13**(2):41–47 DOI: 10.20848/kontyu.13.2_41

Noh YT, Baek KM, Shin, IC, Moon, IH. 1990. Propagation of Korean fireflies, *Luciola lateralis* Motschulsky. *The Korean Journal of Entomology* **20**:1–9.

Novák M. 2017. Redescription of immature stages of central European fireflies, Part 1: *Lampyris noctiluca* (Linnaeus, 1758) larva, pupa and notes on its biology (Coleoptera: Lampyridae: Lampyrinae). *Zootaxa* **4247(4)**:429–444 DOI: 10.11646/zootaxa.4247.4.5

Novák M. 2018a. Redescription of immature stages of central European fireflies, Part 2: *Lamprohiza splendidula* (Linnaeus, 1767) larva, pupa and notes on its life cycle and behaviour (Coleoptera: Lampyridae). *Zootaxa* **4378**(4):516–532 DOI: 10.11646/zootaxa.4378.4.4

Novák M. 2018b. Redescription of immature stages of central European fireflies, Part 3: *Phosphaenus hemipterus* (Goeze, 1777) larva, pupa and notes on its life cycle and behaviour, with a key to three Central European lampyrid larvae (Coleoptera: Lampyridae). *Zootaxa* **4382**(3):450–464 DOI: 10.11646/zootaxa.4382.3.2

Nunes VCDS, Lemos-De-Matos EF, Lima W, Vaz SNC, Mermudes JRM, Silveira LFL. 2021. Lights ahead: morphology and life stages of the spotted tortoise firefly, *Aspisoma sticticum* Gemminger, 1870 – fireflies with a unique extra pair of lanterns on the larval pronotum (coleoptera: lampyridae). *Annales Zoologici* **71**(1):153–178. DOI: 10.3161/00034541ANZ2021.71.1.007

O'Donald P. 1968. Natural selection by glow-worms in a population of *Cepaea nemoralis*. *Nature* **217**:194 DOI: 10.1038/217194a0

Oba Y, Mori N, Yoshida M, Inouye S. 2010. Identification and characterization of a luciferase isotype in the Japanese firefly, *Luciola cruciata*, involving in the dim glow of firefly eggs. *Biochemistry* **49**(51): 10788–10795 DOI:10.1021/bi1016342

Oba Y, Oba Y, Konishi T. 2012. Note on the larval distribution of the terrestrial firefly, *Luciola parvula* Kiesenwetter, 1874, from near the National Composite Center in Nagoya University Higashiyama Campus. *Bulletin of the Nagoya University Museum* **28**:85–88 DOI: 10.18999/bulnum.028.08

Oba Y, Sato M, Ohta Y, Inouye S. 2006. Identification of paralogous genes of firefly luciferase in the Japanese firefly, *Luciola cruciata*. *Gene* **368**:53-60 DOI: 10.1016/j.gene.2005.10.023

Oertel D, Case JF. 1976. Neural excitation of the larval firefly photocyte: slow depolarization possibly mediated by a cyclic nucleotide. *Journal of Experimental Biology* **65**:213–227.

Oertel D, Lindberg KA, Case JF. 1975. Ultrastructure of the larval firefly light organ as related to control of light emission. *Cell and Tissue Research* **164**:27–44. DOI: 10.1007/BF00221693

Oh HS, Kang YK, Nam SH. 2009a. Effect of Water temperature on the climbing up of larvae of firefly, *Luciola lateralis* (Coleoptera: Lampyridae) *Korean Journal of Applied Entomology* **48**(2):203–209 DOI: 10.5656/KSAE.2009.48.2.203

Oh HS, Kang, YK, Nam SH. 2009b. Ecological characteristics of the firefly, *Luciola lateralis* (Coleoptera: Lampyridae). *Korean Journal of Applied Entomology* **48**(2):197–202. DOI: 10.5656/KSAE.2009.48.2.197

Ohba N, Azuma S, Nishiyama K, Goto Y, Suzuki H, Sato Y, Kawashima I. 1994. Morphology, behavior and life cycle of *Luciola owadai* (Coleoptera: Lampyridae). *Science Report of the Yokosuka City Museum* **42:**13–26.

Ohba N, Goto Y, Kawashima I. 1995. Colour and marking patterns of the larval stage in genus *Pyrocoelia* (Coleoptera: Lampyridae) from Japan. *Science Report of the Yokosuka City Museum* **43**:1–9.

Ohba N, Goto Y, Kawashima I. 1996. External morphology, color-making patterns and habitats of the larval stage in genus *Stenocladius* (Coleoptera: Lampyridae). *Science Reports of the Yokosuka City Museum* **44**:21–31.

Ohba N, Goto Y. 1989. Morphology and behaviour of *Luciola yayeyamana* (Coleoptera: Lampyridae). *Science Report of the Yokosuka City Museum* **37**:1–8.

Ohba N, Goto Y. 1991. Morphology and behavior of the firefly, *Pristolycus saguratus*. *Science Report of the Yokosuka City Museum* **39**:1–5.

Ohba N. 1983. Studies on the communication system of Japanese fireflies. *Science Report of Yokosuka City Museum* **30**:1–60.

Ohba N. 1986. Life of a firefly, *Luciola lateralis*. *Insectarium* **23**:156–162.

Ohba N. 1988a. *Gaji firefly*. Tokyo: Bun-ichi Sogo Shuppan.

Ohba N. 1988b. Aquatic glowworms. *Insect and Nature* **23**:8–13.

Ohba N. 1991. Rearing aquatic glowworms in a water tanks as an eco-system. *Insectarium* **28(6):**12–15.

Ohba N. 1997. *Breeding and observation of the fireflies*. Tokyo: Hartshuppan Press.

Ohba N. 2005. Feeding habits of the larvae of *Pyrocoelia abdominalis* and *P. atripennis* (Coleoptera: Lampyridae) to the land snail, *Acusta despecta*. *Science Report of the Yokosuka City Museum* **52**:1–19.

Ohba N. 2007. Feigning death in larvae of the firefly *Pyrocoelia fumosa* (Coleoptera: Lampyridae). *Science Report of the Yokosuka City Museum* **54**:59–65.

Ohba N. Sim SH. 1994. The morphology, behaviour and life cycle of *Pteroptyx valida* (Coleoptera: Lampyridae) in Singapore. *Science Report of the Yokosuka City Museum* **42**:1–11.

Ohtsuki H, Yokoyama J, Ohba N, Ohmiya Y, Kawata M. 2014. Expression of the nos gene and firefly flashing: A test of the nitricoxide-mediated flash control model. *Journal of Insect Science* **14**:56 DOI: 10.1093/jis/14.1.56

Okada YK. 1928. Two Japanese aquatic glowworms. *Transactions of the Entomological Society of London* **76**:101–109. DOI: 10.1111/j.1365-2311.1928.tb01193.x

Owens AC, Lewis SM. 2021. Effects of artificial light on growth, development, and dispersal of two North American fireflies (Coleoptera: Lampyridae). *Journal of Insect Physiology,* **130**(104200):1–8.

Owsjannikow P. 1864. Über das leuchten der larven der *Lampyris noctiluca*. *Bulletin de L'Académie Impériale des Sciences de St.-Pétersbourg* **7**:55–61.

Paiva, CA. **1919**. Notes on the Indian glow-worm ((*Lamprophorus tenebrosus* (Wlk.)). *Records of the Indian Museum* **16(1)**:19–28.

Panigrahi A. 2000. *Lampyris* larva (glowworm), the effective predator of the pestiferous slug *Laevicaulis alte* (Ferussac). *Environment and Ecology* **18(4)**:1011–1013.

Peterson GD. 1957. *Lamprophorus tenebrosus* introduced into Guam to combat the Giant African snail. *Journal of Economic Entomology* **50**:114. DOI: 10.1093/jee/50.1.114

Peterson MK. 1970. The fine structure of the larval firefly light organ. *Journal of Morphology* **131**(1):103–115. DOI: 10.1002/jmor.1051310107

Planet L. 1908. De la larve et de la nymphe du ver-luisant commun (*Lampyris noctiluca* Linn.). *Le Naturaliste* (Paris) **30**:211–213.

Pototskaja VA. 1983. Phylogenetic links and composition of the superfamily Cantharoidea (Coleoptera) based on study of larval characters. *Entomologicheskoe obozrenie* **62**:549–554.

Qin LH, Fu XH. 2009. Observation on predation behavior and functional morphology of larval head in two species of firefly *Diaphanes* sp. and *Pyrocoelia pectoralis*. *Chinese Bulletin of Entomology* **46(1)**:125–128.

Raj JS. 1941. The giant glow-worm of Tambaram. *Madras Christian College Magazine* **11**(2).

Raj JS. 1943a. On the external morphology of the larva of the glow-worm, *Diaphanes* sp. (Lampy: Col.). *Current Science* **12**:276–278.

Raj JS. 1943b. XXII. Observations on a few cases of larval ecdysis of the Indian glow-worm *Lamprophorus tenebrosus* Wlk. *The Journal of the Bombay Natural History Society* **44(1)**:142–143.

Raj JS. **1943c**. On the mouth-parts of the Indian glow-worm, *Lamprophorus tenebrosus* wlk. *Current Science* **12**(3):83–84.

Raj JS. 1947. Two species of undescribed Lampyrid larvae from S. India. Proceedings of the Indian Academy of Science 25:188–194. DOI: 10.1007/BF03049684

Raj JS. 1952. An aquatic glow-worm from Alleppey. *Current Science Bangalore* **21**:222.

Rey MC. 1882. Description de la larve de la *Lamprorhiza mulsanti*. *Annales de la Société Linnéenne de Lyon* **29**:143–145.

Robinson M. 2009. Glow worms attacking snails on Cape York. *Malacological Society of Australasia Newsletter* **135**:1–2.

Rosa SP. 2007. Description of *Photuris fulvipes* (Blanchard) immatures (Coleoptera, Lampyridae, Photurinae) and bionomic aspects under laboratory conditions. *Revista Brasileira de Entomologia* **51(2)**:125–130. DOI: 10.1590/S0085-56262007000200001

Sato N. 2019. Prey-tracking behavior and prey preferences in a tree-climbing firefly. *PeerJ* **7**:e8080 DOI: 10.7717/peerj.8080

Saxton NA, & Powell GS, Serrano AKM, Bybee SM. 2019. Natural history and ecological niche modelling of coastal *Atyphella* Olliff larvae (Lampyridae: Luciolinae) in Vanuatu. *Journal of Natural History* **53**(45-46): 2771–2780 DOI: 10.1080/00222933.2020.1749955

Schaller F, Schwalb H. 1961. Attrappenversuch mit Larven und Imagines heimischer Leuchtkäfer (Lampyrinae). *Verhandlungen der Deutschen Zoologischen Gesellschaft* **1960**:154–166.

Schaller F. 2001. On the glowing and preying behaviour of lampyrid and pyrophorine larvae (Coleoptera: Lampyridae, Elateridae): an open subject for future research in Amazonia - Scientific note. *Amazoniana: Limnologia et Oecologia Regionalis Systematis Fluminis Amazonas* **16(3/4)**:483–486.

Schwalb HH. 1961. Beiträge zur Biologie der einheimischen Lampyriden *Lampyris noctiluca* Geoffr. und *Phausis splendidula* Lec. und experimentelle analyse ihres Beutefang- und Sexualsverhaltens. *Zoologische Jahrbücher, Abteilung für Systematik, Geographie und Biologie der Tiere* **88**:399–550.

Sekine M, Goto M, Ito N, Tanaka K, Kanao M, Inoue T. 2007. Construction of a firefly stream by using a physical habitat evaluation method. *Ecology and Civil Engineering* **10**(2):103–116 DOI 10.3825/ece.10.103

Sivinski J. 1981. The Nature and possible functions of luminescence in Coleoptera larvae. *The Coleopterists Bulletin* **35(2)**:167–179

Sivinski JM, Lloyd JE, Beshers SN, Davis LR, Sivinski RG, Wing SR, Sullivan RT Peterson E. 1998. A natural history of Pleotomodes needhami Green (Coleoptera: Lampyridae): a firefly symbiont of ants. *The Coleopterists Bulletin* **52(1)**:23–30.

Smedley SR, Risteen RG, Tonyai KK, Pitino JC, Hu Y, Ahmed ZB, Christofel BT, Gaber M, Howells NR, Mosey CF, Rahim FU, Deyrup ST. 2017. Bufadienolides (lucibufagins) from an ecologically aberrant firefly (*Ellychnia corrusca*). *Chemoecology* **27**:141–153 DOI: 10.1007/s00049-017-0240-6

Sodeman Jr WA, Rodrick GE, Vincent AL. 1980. Lampyridae larva: a natural predator of schistosome vector snails in Liberia. *The American Journal of Tropical Medicine and Hygiene* **29**(2):319 DOI: 10.4269/ajtmh.1980.29.319

Stansbury MS, Moczek AP. 2014. The function of Hox and appendage-patterning genes in the development of an evolutionary novelty, the *Photuris* firefly lantern. *Proceedings of the Royal Society B* **281**(1782):20133333 DOI: 10.1098/rspb.2013.3333

Strause LG, Case JF. 1981. Neuro-pharmacological studies on firefly light organs during metamorphosis. *Journal of Insect Physiology* **27**(1):5–15 DOI: 10.1016/0022-1910(81)90025-1

Strause LG, DeLuca M, Case JF. 1979. Biochemical and morphological changes accompanying light organ development in the firefly, *Photuris pennsylvanica. Journal of Insect Physiology* **25**(4), 339–347 DOI: 10.1016/0022-1910(79)90022-2

Strause LG, DeLuca M. 1981. Characteristics of luciferases from a variety of firefly species: evidence for the presence of luciferase isozymes. *Insect Biochemistry* **11**(4):417–422 DOI: 10.1016/0020-1790(81)90075-5

Tabaru Y, Kouketsu T, Oba M, Okafuji S. 1970. Effects of some organophosphorus insecticides against the larvae of Genji firefly, *Luciola cruciata* and their prey, Japanese melanial snail *Semisulcospira bensoni*. *Medical Entomology and Zoology* **21**:178–181.

Takeda M, Amano T, Katoh K, Higuchi H. 2006. The habitat requirement of the Genji-firefly *Luciola cruciata* (Coleoptera: Lampyridae), a representative endemic species of Japanese rural landscapes. *Biodiversity and Conservation* **15**:191–203 DOI: 10.1007/s10531-004-6903-y

Thancharoen A, Ballantyne LA, Branham MA, Jeng ML. 2007. Description of *Luciola aquatilis* sp. nov., a new aquatic firefly (Coleoptera: Lampyridae: Luciolinae) from Thailand. *Zootaxa* **1611**:55–62 DOI: 10.11646/zootaxa.1611.1.4

Tisi LC, De Cock R, Stewart AJA, Booth D, Day JC. 2014. Bioluminescent leakage throughout the body of the glow-warm Lampyris noctiluca (Coleoptera: Lampyridae). *Entomologia Generalis* **35**(1):47–51 DOI: 10.1127/0171-8177/2014/0003

Tonolli PN, Okawachi FM, Abdalla FC, Viviani VR. 2011. Bioluminescent fat body of larval *Aspisoma lineatum* (Coleoptera: Lampyridae) firefly: ontogenic precursor of lantern's photogenic tissue. *Annals of the Entomological Society of America* **104**(4):761–767 DOI: doi.org/10.1603/AN10143

Tozzetti AT. 1866. Come sia fatto l'organo che fa lume nella Lucciola Volante dell'Italia centrale (Luciola Italica) e come le fibre muscolari in questo ed altri insetti ed artropodi: osservazioni. *Memorie della Società italiana di scienze naturali* **Tomo I**(8):1–27.

Tozzetti AT. 1870. Sull'organo che fà lume nelle Luccioloe volante d'Italia (*Luciola italica*). Nuove osservazioni. *Bolletino della Società Entomológica Italiana* **2**:177–189.

Trice E, Tyler J, Day JC. 2004. Description of pleural defensive organs in three species of firefly larvae (Coleoptera, Lampyridae). *Zootaxa* **768**:1–11 DOI: 10.11646/zootaxa.768.1.1

Trice E, Tyler J. 2007. The structure of the larval tail organ in the European glow-worm, *Lampyris noctiluca* (Coleoptera: Lampyridae). *Quekett Journal of Microscopy* **40(5)**:417–422.

Tyler J, Trice E. 2001. A description of a possible defensive organ in the larvae of the European Glow-worm *Lampyris noctiluca* (Linnaeus) (Lampyridae). *The Coleopterist* **10(3)**:75–78.

Tyler J, McKinnon W, Lord GA, Hilton PJ. 2008. A defensive steroidal pyrone in the glow-worm *Lampyris noctiluca* L. (Coleoptera: Lampyridae). *Physiological Entomology* **33**(2):167–170 DOI: 10.1111/j.1365-3032.2007.00610.x

Tyler J. 1997a. Rearing the Glow-worm *Lampyris noctiluca* Linnaeus (Lampyridae). *The Coleopterist* **5(3)**:77.

Tyler J. 1997b. A two-headed larva of the glow-worm *Lampyris noctiluca* Linnaeus (Lampyridae). *Coleopterist* **6**(2):67.

Tyler J. 2001a. Are glow-worms *Lampyris noctiluca* (Linnaeus) (Lampyridae) distaestful? *The Coleopterist* **9(3)**:148.

Tyler J. 2001b. A previously undescribed defence mechanism in the larval glow-worm *Lampyris noctiluca* (Linnaeus)? *The Coleopterist* **10(2)**:38

Tyler J. 2002. *Glow-worms*. Sevenoaks: Tyler-Scagell.

Underwood TJ, Tallamy DW, Pesek JD. 1997. Bioluminescence in firefly larvae: a test of the aposematic display hypothesis (Coleoptera: Lampyridae). *Journal of Insect Behavior* **10**:365–370 DOI: 10.1007/BF02765604

Vaz S, Silveira LFL, Rosa SP. 2020. Morphology and life cycle of a new species of *Psilocladus* Blanchard, 1846 (Coleoptera, Lampyridae, Psilocladinae), the first known bromeliad-inhabiting firefly. *Papéis Avulsos de Zoologia* **60**(spe):e202060(s.i.)24 DOI: 10.11606/1807-0205/2020.60.special-issue.24

Vaz SNC, Guerrazzi MC, Rocha M, Faust LF, Gabriel Khattar G, Mermudes JRM, Silveira LFL. 2021. On the intertidal firefly genus *Micronaspis* Green, 1948, with a new species and a phylogeny of Cratomorphini based on adult and larval traits (Coleoptera: Lampyridae). *Zoologischer Anzeiger*, **292:** 64–91.

Vencl FV, Shah S, Gerber A, Carlson AD. 2012. Octopamine and DUM neurons orchestrate the larval firefly aposematic defense. *In* Kirton LG, Day JC, Lim GT. (ed). *Lampyrid: Volume 2 2012: The Journal of Bioluminescent Beetle Research (Lampyrid Journal)*. Oxfordshire: Brazen Head Publishing, 99–112.

Verhoeff K. 1924. Zur Biologie der Lampyriden. *Zeitschrift für wissenschaftliche Insektenbiologie* **19**:79–145.

Viviani VR, Bechara EJH. 1995. Bioluminescence of Brazilian fireflies (Coleoptera: Lampyridae): spectral distribution and PH effect on luciferase-elicited colors. Comparison with elaterid and phengodid luciferases. *Photochemistry and Photobiology* **62**(3): 490–495. DOI: 10.1111/j.1751-1097.1995.tb02373.x

Viviani VR, Okawachi FM, Scorsato V, Abdalla FC. 2008. CCD imaging of basal bioluminescence in larval fireflies: clues on the anatomic origin and evolution of bioluminescence. *Photochemical and Photobiological Sciences* **7**(4):448–452 DOI: 10.1039/B718016K

Viviani VR, Arnoldi FGC, Brochetto-Braga M, Ohmiya Y. 2004. Cloning and characterization of the cDNA for the Brazilian *Cratomorphus distinctus* larval firefly luciferase: similarities with European *Lampyris noctiluca* and Asiatic *Pyrocoelia* luciferases. *Comparative Biochemistry and Physiology, Part B* **139(2)**:151–156.

Viviani VR. 2001. Fireflies (Coleoptera: Lampyridae) from Southeastern Brazil: habitats, life history, and bioluminescence. *Annals of the Entomological Society of America* **94**(1): 129–145 DOI: 10.1603/0013-8746(2001)094[0129:FCLFSB]2.0.CO;2

Vivianti VR. Rosa SP, Martins MA. 2012. *Aspisoma lineatum* (Gyllenhal) (Coleoptera: Lampyridae) firefly: description of the immatures, biological, and ecological aspects. *Neotropical Entomology* **41**:89–94 DOI: 10.1007/s13744-011-0006-8

Vogel R. 1912. Beiträge zur anatomie und biologie der larve von *Lampyris noctiluca*. *Zoologischer Anzeiger* **39(17/18)**:515–519.

Vogel R. 1915. Beitrag zur Kenntnis des Baues und der Lebensweise der Larve von *Lampyris noctiluca. Zeitschrift fûr Wissenschaftliche Zoologie* **112**:291–432.

Vogel R. 1922 Uber die Topographie der Leuchtorgane von *Phausis splendidula* Leconte. *Biologisches Zentralblatt* **42**:138–140.

Vongsangnak W, Chumnanpuen P, Sriboonlert A. 2016. Transcriptome analysis reveals candidate genes involved in luciferin metabolism in *Luciola aquatilis* (Coleoptera: Lampyridae). *PeerJ* **4**:e2534 DOI: 10.7717/peerj.2534

Wang Y, Fu X, Lei C, Jeng ML, Nobuyoshi O. 2007. Biological characteristics of the terrestrial firefly *Pyrocoelia pectoralis* (Coleoptera: Lampyridae) *Coleopterists Bulletin* **61**(1):85–93

Wenzel HW. 1896. Notes on Lampyridae, with the description of a female and larva. *Entomological News* **7**:294–296.

Wickham HF. 1895. On the larvae of *Lucidota*, *Sinoxylon*, and *Spermophagus*. *Bulletin from the Laboratories of Natural History of the State University of Iowa* **3(3)**:31–35.

Wielowiejski HR. 1882. Studien uber die Lampyriden. Zeitschrift für wissenschaftliche Zoologie **37**:354–428.

Wienhausen G, DeLuca M. 1985. Luciferases from different species of fireflies are antigenically similar. *Photochemistry and Photobiology* **42**(5):609–611 DOI: 10.1111/j.1751-1097.1985.tb01619.x

Wijekoon WMCD, Wegiriya HCE, Bogahawatta CNL. 2016. Predatory role of lampyrid larvae (*Lamprigera tenebrosa*); laboratory experiments to control agricultural molluscan pests, *Achatina fulica* & *Laevicaulis altae. International Journal of Science, Environment and Technology* **5**:1–6.

Wilcox A, Lewis S. 2019. Fluorescence in fireflies (Coleoptera: Lampyridae): using sentinel prey to investigate a possible aposematic signal. *Florida Entomologist* **102**(3):614–618 DOI 10.1653/024.102.0342

Williams F. X. 1917b. Photogenic organs and embryology of some lampyrids. *Journal of Morphology* **28(1)**:145–207.

Williams FX. 1914. Prothetely in the larva of *Photuris pennsylvanica* De Geer. *Psyche* **21**:126–129.

Williams FX. 1917a. Notes on the life-history of some North American Lampyridae. *Journal of the New York Entomological Society* **25(1)**:11–33.

Wootton A. 1976. Rearing the glow-worm, *Lampyris noctiluca* L. (Coleoptera: Lampyridae). The Entomologist's Record & Journal of Variation **88**: 64–67.

Wu C-H, Ho J-Z, Jeng M-L, Yang P-S. 2012. The survey of the firefly resources in Chihnan National Forest Recreation area and the fireflies' resource utilization. *Formosan Entomologist* **32**: 249–269 DOI:10.6661/TESFE.2012015

Wu C-H, Yang P-S. 2008. Survey of the firefly resources in Tungshih Forest. *Formosan Entomologist* **28**(3):195–209 DOI: 10.6661/TESFE.2008015.

Wu W-C, Perng JJ. 2007. Habitat environmental factors and population fluctuations of the firefly, *Pyrocoelia analis* (Coleoptera: Lampyridae). *Formosan Entomologist* **27**(1):31–45. DOI: 10.6661/TESFE.2007003

Wunsch E. 1995. Die Larvalentwicklung von *Lampyris noctiluca* (L.) im Naturschutzgebiet Federsee (Coleoptera: Lampyridae). *Mitteilungen des Internationalen Entomologischen Vereins e.V. Frankfurt a.M.* **20(1/2)**:1–14.

Wynberg H, Meijer EW, Hummelen JC, Dekkers HPJM, Schippers PH, Carlson AD. 1980. Circular polarization observed in bioluminescence. *Nature* **286**:641–642. DOI: 10.1038/286641a0

Xian LM, YU WS, Cao M, Cao CQ. 2011. The Life cycle and behaviour of *Lamprigera yunnana* (Fairmaire). *Jiangxi Plant Protection* **2011(1)**:22–24

Yajima M. 2007. Breeding fireflies at Tama Zoo: an ecological approach. *Der Zoologische Garten* **77**(2):84–95 DOI: 10.1016/j.zoolgart.2007.07.003

Yajima M. 2015. Forty-year efforts to establish firefly populations at the Imperial Palace: polymorphic patterns in larval development and life cycle. *Japanese Journal of Entomology New Series* **18**(4):106–117.

Yang XJ, Zheng HL, Liu YY, Li HW, Jiang YH, Lin LB, Deng XY, Zhang QL. 2020 Selection of reference genes for quantitative real-time PCR in *Aquatica leii* (Coleoptera: Lampyridae) under five different experimental conditions. *Frontiers in Physiology* **11**: 555233 DOI: 10.3389/fphys.2020.555233.

Yeh S. 1999. *The habitat management and food preference of an aquatic firefly, Luciola ficta (Coleoptera: Lampyridae).* Master's Thesis. Graduate Institute of Plant and Entomology, National Taiwan University, Taiwan.

Yiu V. 2011. Observations on the luminescence configurations of eight firefly genera and their immature stages. *Hong Kong Entomological Bulletin* **3**(1):20–30.

Yoshida T, Ujiie R, Savitzky AH, Jono T, Inoue T, Yoshinaga N, Aburaya S, Aoki W, Takeuchi H, Ding L, Chen Q, Cao C, Tsai T-S, Silva A, Mahaulpatha D, Nguyen TT, Tang Y, Mori N, Mori A. 2020. Dramatic dietary shift maintains sequestered toxins in chemically defended snakes. *Proceedings of the National Academy of Sciences of the United States of America* **117**(11):5964–5969 DOI 10.1073/pnas.1919065117

Yuan HH, Fu XH, Zhang Y, Zheng XL, Lei CL. 2007. The ultrastructure of light organs in adults and larvae of the firefly, *Luciola substriata*. *Chinese Bulletin of Entomology* **44(3)**:409–414.

Yuma M. 1981. The body size variations of the climbing larvae of the firefly, *Luciola cruciata* (Coleoptera; Lampyridae). *Japanese Journal of Ecology* **31**(1):57–66 DOI 10.18960/seitai.31.1_57

Yuma M. 1982. The climbing larvae of *Luciola cruciata*. *Insectarium* **19(5)**:14–22.

Yuma M. 1984. Egg size and viability of the firefly *Luciola cruciata* (Coleoptera: Lampyridae). *Japanese Journal of Entomology* **52**:615–629.

Yuma M. 1986. Growth and size variations in the larvae of *Luciola cruciata* (Coleoptera: Lampyridae) in relation to the egg size. *Physiology and Ecology Japan 23:45–78.*

Yuma M. 2007. Effect of rainfall on the long-term population dynamics of the aquatic firefly *Luciola cruciata*. *Entomological Science* **10**(3):237–244 DOI: 10.1111/j.1479-8298.2007.00219.x

Zaragoza-C S, Cifuentes-R P, Domínguez-L DE, González-R M, Ishwari G. Gutiérrez-C IG, López-P S, Rodríguez-M GM, Vega-B V, Zurita-G ML. 2020. Proyecto "Luciérnagas de México". *Boletín de la AMXSA* **4(1)**:20–22.

Zhang Q-L, Guo J, Deng X-Y, Wang F, Chen, J-Y, Lin L-B. 2019. Comparative transcriptomic analysis provides insights into the response to the benzo(a)pyrene stress in aquatic firefly (*Luciola leii*). *Science of the Total Environment* **661**:226–234 DOI 10.1016/j.scitotenv.2019.01.156

Zhang Q-L, Jiang Y-H, Dong Z-X, Li H-W, Lin L-B. 2021. Exposure to benzo[a]pyrene triggers distinct patterns of microRNA transcriptional profiles in aquatic firefly *Aquatica wuhana* (Coleoptera: Lampyridae). *Journal of Hazardous Materials* **401**:123409 DOI: 10.1016/j.jhazmat.2020.123409

Zhang Q-L, Li H-W, Dong Z-X, Yang X-J, Lin L-B, Chen J-Y, Yuan M-L. 2020. Comparative transcriptomic analysis of fireflies (Coleoptera: Lampyridae) to explore the molecular adaptations to fresh water. *Molecular Ecology* **29**(14): 2676–2691 DOI: 10.1111/mec.15504

Zheng X, Fu X, Zhang S, Lei C. 2008. Larval behavior probably associated with respiration in Luciola substriata Gorham (Coleoptera: Lampyridae) *Coleopterists Bulletin* **62**(4):550–559 DOI: 10.1649/1053.1

Zheng X-L, Lu C, Fu X-H, Lei C-L. 2008a. Effects of water quality in urban waters of Wuhan on the larval respiratory behavior of *Luciola leii*. *Kunchong Zhishi* **45**(6):953–957.

Zheng XL, Yuan HH, Wang YY, Fu XH, Lei CL. 2008b. Respiratory system and respiratory behaviors of *Luciola leii* larvae. *Chinese Bulletin of Entomology* **45(3)**:445–448.
